# Supplementary material for: Comprehensive marine substrate classification applied to Canada’s Pacific shelf
Source: PLoS One. 2021 Oct 29;16(10):e0259156. doi: 10.1371/journal.pone.0259156 (PMC8555849; doi:10.1371/journal.pone.0259156)
Supplement: S1 File — (DOCX) [file pone.0259156.s001.docx]

# Comprehensive, coastwide marine substrate classification Supporting Information

2021/10/15

**S1 – Performance metrics**

We used a diversity of metrics to evaluate the performance of the random forest models. These metrics are calculated from a confusion, or error matrix [1, 2], which describes how well the mapped, or predicted values (represented as rows) correspond with the reference, or validation data (represented as columns).

We assess both model accuracy and model error. Measures of accuracy commonly compare predictions to a no-information baseline (i.e., improved performance over random guessing). We use the True Skill Statistic [TSS, 3], Normalized Accuracy, and the True Negative Rate (TNR) to provide a complete picture of model performance. Accuracy and TNR range from 0 to +1, while the TSS values range from -1 to +1. When predictions are binary (e.g., predictions of species presence or absence) the no-information baseline for Accuracy and TNR is 0.5, but this must be adjusted when multiple classes are used. In our case, with 4 classes, the no-information baseline for Accuracy is 0.25, while for correct absence prediction (TNR) it is 0.75. The TSS is corrected for random performance [3, 4] and integrates accuracy across all classes so it’s no-information baseline is always 0.

To compare model performance across classes we use the class-based metrics commonly termed Producer and User Accuracies [2]. Producer's Accuracy is the probability that a given reference class is correctly predicted. This describes accuracy from the point of view of the map maker. It is calculated by column, with the number of correctly classified reference units divided by the class sample size. User's Accuracy represents accuracy from the point of view of a map user. It is the proportion of a particular class correctly predicted, and is calculated as the number of correct predictions for a class divided by the row total. We adopt the term Reliability (R) proposed by Congalton [1], as it represents the probability of a class prediction matching a field observation.

Recognizing the importance of sample prevalence, we introduce Imbalance (I) as an aggregated metric to more easily compare across data sets. It is defined as the sum of the sample size differences between all classes, divided by the total pairwise comparisons. A perfectly balanced dataset would have equal prevalence of all classes and an imbalance score of 0.

We assess model error following the work of Pontius and colleagues [e.g., 5, 6], who propose a set of error metrics to describe the error or mismatch between the predicted and reference data. Quantity error measures the difference in prevalence, and Exchange and Shift describe how classes are misassigned. The goals of these metrics are to 1) show deficiencies in agreement between predicted and reference data (rather than how much predictions are better than random) [5], and 2) to separate a map’s information of Quantity (i.e., matching of prevalence) from the information it provides on location [7]. Pontius and Millones [5] argue that ‘less than perfect’ is more relevant compared to ‘better than random’ (as measured by Kappa and TSS). These class-based metrics can also be calculated as integrated scores [5].

**S2. Reproducing the analysis**

The source data for this analysis include observational data (as points) and predictor data (as rasters). Polygons outlining the boundaries of the 20 m regional models were created and used to partition the coastwide build data by region for tests of stationarity and resolution effects on performance. These source data are provided as RData files packaged with the analytic code. The RData files include the build and independent data sets described in the main text, with the 20 m and 100 m predictor variables attached. This allows the analysis to be reproduced. However, the study area-wide predictions will not be reproduced because several of the predictor layers require data licensing agreements. Please contact the authors to arrange use of these data.

Point data were extracted from various Fisheries and Oceans (DFO) databases by SD and JL. The development of the predictor rasters was led by CF. EG contributed the Fetch layers. The R code was developed by CF and EG.

Additional details are provided in the READ.ME file associated with the R project (https://github.com/ejgregr/substrate_model).

**R libraries used**

caret [8]: Contingency table and metrics
Colorbrewer [9]: Color selection including color-blind palettes
diffeR [10]: Error metrics
dplyr [11]: Data manipulation functions
e1071 [12]: Miscellaneous functions
forcats [13]: Tools for categorical variables
ggplot2 [14]: Bar plots
measures [15]: Performance measures
PresenceAbsence [16]: Presence-absence model analysis
ranger [17]: Random forest implementation
raster [18]: Geographic Data Analysis and Modeling
reshape2 [19]: Shaping data for ggplot2
rgdal [20]: Geospatial package
stringr [21]: String operations
superheat [22]: Heat maps
tidyr [23]: Data manipulation functions
vegan [24]: Community ecology

**References**

1. Congalton RG. A review of assessing the accuracy of classifications of remotely sensed data. Remote Sens Environ. 1991;37(1):35-46.

2. Guisan A, Zimmermann NE. Predictive habitat distribution models in ecology. Ecol Model. 2000;135:147-86.

3. Allouche O, Tsoar A, Kadmon R. Assessing the accuracy of species distribution models: Prevalence, kappa and the true skill statistic (TSS). J Appl Ecol. 2006;43:1223-32. doi: 10.1111/j.1365-2664.2006.01214.x. PubMed PMID: 6607.

4. Jolliffe IT, Stephenson DB. Forecast verification: a practitioner's guide in atmospheric science: John Wiley & Sons; 2012.

5. Pontius RGJ, Millones M. Death to Kappa: birth of quantity disagreement and allocation disagreement for accuracy assessment. Int J Remote Sens. 2011;32(15):4407-29.

6. Pontius RGJ, Santacruz A. Quantity, exchange, and shift components of difference in a square contingency table. Int J Remote Sens. 2014;35(21):7543-54.

7. Pontius RGJ, Thontteh O, Chen H. Components of information for multiple resolution comparison between maps that share a real variable. Environ Ecol Stat. 2008;15:111-42.

8. Kuhn M. caret: Classification and Regression Training. 2020. R package version 6.0-86. Available from: <https://CRAN.R-project.org/package=caret>.

9. Neuwirth E. RColorBrewer: ColorBrewer Palettes. 2014. R package version 1.1-2. Available from: <https://CRAN.R-project.org/package=RColorBrewer>.

10. Pontius Jr RG, Santacruz A. diffeR: Metrics of Difference for Comparing Pairs of Maps or Pairs of Variables. 2019. R package version 0.0-6. Available from: <https://CRAN.R-project.org/package=diffeR>.

11. Wickham H, Francois R, Henry L, Müller K. dplyr: A Grammar of Data Manipulation. 2020. R package version 1.0.0. Available from: <https://CRAN.R-project.org/package=dplyr>.

12. Meyer D, Dimitriadou E, Hornik K, Weingessel A, Leisch F. e1071: Misc Functions of the Department of Statistics, Probability Theory Group (Formerly: E1071), TU Wien. 2019. R package version 1.7-3. Available from: <https://CRAN.R-project.org/package=e1071>.

13. Wickham H. forcats: Tools for Working with Categorical Variables (Factors). 2020. R package version 0.5.0. Available from: <https://CRAN.R-project.org/package=forcats>.

14. Wickham H. ggplot2: Elegant graphics for data analysis. Springer-Verlag, New York, USA, pp. 260. 2016.

15. Probst P. measures: Performance Measures for Statistical Learning. 2018. R package version 0.2. Available from: <https://CRAN.R-project.org/package=measures>.

16. Freeman EA, Moisen G. PresenceAbsence: An R package for presence absence analysis. Journal of Statistical Software 23 (11): 31 p. 2008.

17. Wright MN, Ziegler A. ranger: A fast implementation of random forests for high dimensional data in C++ and R. arXiv preprint arXiv:150804409. 2015.

18. Hijmans RJ. raster: Geographic Data Analysis and Modeling. 2020. R package version 3.3-13. Available from: <https://CRAN.R-project.org/package=raster>.

19. Wickham H. Reshaping data with the reshape package. Journal of statistical software. 2007;21(12):1-20.

20. Bivand R, Keitt T, Rowlingson B. rgdal: Bindings for the ‘Geospatial’ Data Abstraction Library. 2020. R package version 1.5-12. Available from: <https://CRAN.R-project.org/package=rgdal>.

21. Wickham H. stringr: Simple, Consistent Wrappers for Common String Operations. 2019. R package version 1.4.0. Available from: <https://CRAN.R-project.org/package=stringr>.

22. Barter R, Yu B. superheat: A Graphical Tool for Exploring Complex Datasets Using Heatmaps. 2017. R package version 0.1.0. Available from: <https://CRAN.R-project.org/package=superheat>.

23. Wickham H, Henry L. tidyr: Tidy Messy Data. 2015. R package version 1.1.0. Available from: <https://CRAN.R-project.org/package=tidyr>.

24. Oksanen J, Blanchet FG, Friendly M, Kindt R, Legendre P, McGlinn D, et al. vegan: Community Ecology Package. 2019. R package version 2.5-6. Available from: <https://CRAN.R-project.org/package=vegan>.

25. Bureau D. Geoduck (*Panope generosa*) density and biomass estimates in Pacific Fishery Management Area 23. Can. Manuscr. Rep. Fish. Aquat. Sci. 3111. 2017; vi + 17p.

26. DFO. Review of Dive Survey Methods for Northern Abalone in British Columbia. Can. Sci. Advis. Sec. 2016/044. 2016.

27. Duprey NMT, Stanton LM. Biomass estimates for sea cucumber (*Parastichopus californicus*, *Cucumaria miniata*, *C. pallida*) as determined through surveys conducted June 2013 to May 2014. Can. Manuscr. Rep. Fish. Aquat. Sci. 3112. 2018; x + 72 p.

28. Leus D, Campbell A, Merner E, Hajas WC, Barton LL. Framework for estimating quota options for the red sea urchin (*Strongylocentrotus franciscanus*) fishery in British Columbia using Shoreline length and linear density estimates. Can. Sci. Advis. Sec. Res. Doc. 2013/094. 2014; vi + 68 p.

29. Waddell B, Zhang Z, Perry RI. Stock assessment and quota options for the green sea urchin, *Strongylocentrotus droebachiensis*, fishery in British Columbia, 2010-2013. Can. Sci. Advis. Sec. Res. Doc. 2010/027. 2010; vi + 36 p.

30. Gregr EJ, Lessard J, Harper J. A spatial framework for representing nearshore ecosystems. Prog Oceanogr. 2013;115:189-201.

31. Haggarty DR, Flemming R, Cooke K, Deleys N, Yamanaka KL. Remotely operated vehicle surveys of rockfish conservation areas in British Columbia, February 2009 – July 2011. Can. Tech. Rep. Fish. Aquat. Sci. 3189. 2017; vi + 141.

32. Davies SC, Gregr EJ, Bureau D, Wills P. Coastal digital elevation models integrating ocean bathymetry and land topography for marine ecological analyses in Pacific Canadian waters. Can. Tech. Rep. Fish. Aquat Sci. 3321. 2019; vi + 38 p.

33. Carignan K, Eakins B, Love M, Sutherland M, McLean S. Bathymetric digital elevation model of British Columbia, Canada: procedures, data sources, and analysis. NOAA National Geophysical Data Center (NGDC). 2013.

34. Gregr EJ. BC_EEZ_100m: A 100 m raster of the Canadian Pacific exclusive economic zone. SciTech Environmental Consulting. Vancouver BC. 2012.

35. Nephin J, Gregr EJ, St. Germain C, Fields C, Finney JL. Development of a species distribution modelling framework and its application to twelve species on Canada’s Pacific coast. Can. Sci. Advis. Sec. Res. Doc. 2020/004. 2020; xii + 107 p.

36. Walbridge S, Slocum N, Pobuda M, Wright DJ. Benthic terrain modeler (BTM) 3.0, tools for understanding and classifying the benthic environment. 2018. Available from: <https://repository.oceanbestpractices.org/handle/11329/1196>.

37. Parks Canada. BCMCA_ECO_Physical_HighRugosity_DATA. British Columbia Marine Conservation Analysis. Vancouver, BC. 2009.

38. Du Preez C. A new arc–chord ratio (ACR) rugosity index for quantifying three-dimensional landscape structural complexity. Landscape Ecol. 2015;30(1):181-92.

39. ESRI. ArcGIS. 2019. 10.4. Available from: <https://www.arcgis.com/index.html>.

40. Masson D, Fine I. Modeling seasonal to interannual ocean variability of coastal British Columbia. Journal of Geophysical Research: Oceans. 2012;117(C10):C10019:1-14. doi: 10.1029/2012jc008151.

41. Soontiens N, Allen SE, Latornell D, Le Souëf K, Machuca I, Paquin J-P, et al. Storm surges in the Strait of Georgia simulated with a regional model. Atmosphere-Ocean. 2016;54(1):1-21.

42. Burrows MT. Influences of wave fetch, tidal flow and ocean colour on subtidal rocky communities. Mar Ecol Prog Ser. 2012;445:193-207.

43. Davies SC, Bureau D, Lessard J, Taylor S, Gillespie GE. Benthic habitat mapping surveys of eastern Haida Gwaii and the north coast of British Columbia, 2013-2015. Can. Tech. Rep. Fish. Aquat. Sci 3278. 2018; vi + 24 p.

*Table S1: Data sampling and preparation details. Sampling and preparation of the substrate observations contained in the build data, compiled from Fisheries and Oceans Canada (DFO) the Canadian Hydrographic Service (CHS) and Natural Resources Canada (NRCan). All data were used in both the 20 and 100 m resolution models except as noted.*

| **Data set** | **Sampling and preparation details** |
| --- | --- |
| DFO Dive | We extracted observations of bottom type collected during DFO invertebrate stock assessment dive surveys (for abalone, red and green sea urchins, red sea cucumber, and geoduck clams) from 13,522 transects surveyed between 1992 and 2015 [25-29].  Substrate type was recorded at quadrats spaced along transects extending from the high water line to 20 m depth. To spatialize the data, quadrat observations were pooled for each transect according to correspondence between recorded dive depth gauge and a predefined depth range because a large portion of the data do not geolocate the quadrats. A Python script was used to position the points in the middle of a pre-defined depth zone at the point closest to the transect origin. We used the depth zones defined by Gregr et al. [30]. Data were pre-processed to exclude points occurring on land. |
| CHS Remotely operated Vehicle (ROV) | We extracted bottom type from 424 ROV rockfish surveys conducted between 2009 and 2011. Continuous observations along transects were classified into points by selecting one point randomly for every 20 m of a transect line [31]. These surveys were limited to the West Coast Vancouver Island, Queen Charlotte Sound, and Strait of Georgia regions. |
| CHS Grabs | Grab data have been historically collected alongside depth sounding data by CHS for over a century (the first salt-water survey in British Columbia was conducted in 1891). Data collected in the second half of the 20^th^ century were recorded on mylar field sheets, which were then transcribed into marine charts. In the early 21^st^ century, CHS digitized the field sheets providing a wealth of high resolution bathymetric and bottom type data. CHS collected and made these data available to DFO Science Branch. Sampling methods range from historic lead-line samples to present-day mechanical grabs. These data collected with a higher density in shallow, hard bottom waters in support of the CHS’s mandate of ensuring safe navigation. |
| CHS Marsh | We included marsh locations to increase the observations of soft substrate, and improve the balance across substrate classes. Marsh locations are documented on bathymetric charts throughout the Canadian Pacific coastal zone. The data were extracted by CHS from their S-57 series digital charts. |
| NRCan Grabs | We included grab samples from NRCan in the 100 m model to improve its performance at depth, and to offset the hard bias in the CHS Grab data. These data were omitted from the 20 m models because the spatial uncertainty exceeds 20 m. |

*Table S2: Predictor data preparation details. All predictors were used to build both the 20 and 100 m resolution random forest models except fetch, which was not used in the 100 m coastwide model.*

| **Data set** | **Preparation details** |
| --- | --- |
| Bathymetry | For the regional models, we used the 20 m bathymetries produced by Davies et al. [32]. The 100 m bathymetry was derived from Carignan et al. [33] and Gregr [34] by Nephin et al. [35].  The 20 m bathymetry models extend 5 km inland to allow changes in elevations in the intertidal region to be characterized accurately. See Davies et al. [32] for more details. |
| Bathymetric derivatives | We created slope, standard deviation of slope, curvature, and bathymetric position index (BPI) predictors from the bathymetry rasters at both resolutions using a Python script that applied functions from the Benthic Terrain Modeller toolbox [36]. First, the depth layers were multiplied by -1 to change positive depth values to negative. These layers were used to generate the derivatives. Initial investigations of the bathymetric derivatives identified artefacts in the data (**Fig. S3**). We therefore smoothed the 20 m bathymetry using the average within a circular neighbourhood with a 3 cell radius before generating slope, standard deviation of slope, curvature, and rugosity. The same derivatives (except rugosity) were generated from the 100 m bathymetry, averaged using a 5 cell radius. |
| Rugosity | We used a high rugosity layer from the British Columbia Marine Conservation Atlas [37] for our 100 m coastwide model due to the scale and coarser resolution. This layer was derived from a 75 m bathymetry with high rugosity defined as the top quantile (20%) of continuous rugosity values. We converted the rugosity polygon to raster and aligned it with our other predictor layers.  For the 20 m regional models, we calculated rugosity using the arc-chord ratio (ACR) method [38]. This method is preferred over surface ratio rugosity calculations because it decouples rugosity from slope, reducing the correlation between these two predictors. |
| Slope and curvature | We calculated slope in degrees, and standard deviation of slope using a 3 x 3 cell rectangular neighbourhood. Curvature, defined as the slope-of-the-slope, identifies a surface as concave or convex. Areas of moderate relief typically have values ranging from -0.5 to 0.5 while areas of extreme relief will vary between -4 and 4 [39]. |
| Bathymetric position index (BPI) | Since BPI is a neighbourhood metric, we used the un-smoothed bathymetry rasters to calculate these layers. BPI values represent a cell’s position relative to an annulus-shaped neighbourhood defined by an inner and outer radius. It is correlated with the neighbourhood size, so for each model resolution we calculated BPI at three scales to capture both small benthic features, and larger trends in terrain.  For the regional models, we calculated fine, medium and broad BPI using annuli of 60 to 500 m, 200 to 2,000 m, 500 to 5,000 m respectively. We used neighborhoods of 300 to 2,500 m, 1,000 to 10,500 m , and 2,500 to 25,000 m for the coastwide model. All BPI layers were standardized using the BTM toolbox. |
| Mean summer tidal and ocean circulation speeds | Two ocean energy layers representing bottom current speeds (metres per second) were derived from a Regional Ocean Modelling System (ROMS) circulation model of Canada’s Pacific coast [40] by Nephin et al. [35], who averaged current velocities to represent different temporal components. General ocean circulation patterns were based on 15-day mean velocities, while tidal current speed was resolved using 3-hour mean velocities. The ROMS model used a 3 by 3 km grid, which was interpolated using Spline with Barriers [39] to create 1 km x 1 km raster layers for mean summer circulation. These layers were resampled to the 100 m and 20 m resolution rasters used in this study.  A circulation model with 440 m x 500 m resolution [41] was used for the 20 m SOG model. Model points were first interpolated using Spline with Barriers [39] to a 40 m cell resolution (because of computational limitations) and then resampled to 20 m using bilinear interpolation. The SOG tidal and circulation layers were created using the same temporal averaging as the ROMS model, and smoothed using Focal Statistics with a 13 cell neighbourhood. |
| Fetch | Defined as the distance over which wind-driven waves can build, fetch provides a proxy of exposure to wave action [42]. We created points every 50 m along the coastline, and estimated exposure for each point using 200 km bearing lines created every 5 degrees. The 72 bearing lines for each point were clipped using the CHS high water coastline and summed. This was done individually for each of the five regions, generating over 750,000 points. These points were then interpolated to build rasters for each region, with slightly different methods.  For highly exposed regions (WCVI and the West coast of HG), we filled the offshore extent with the maximum fetch length before interpolation using natural neighbour [39]. For the Strait of Juan de Fuca portion of the SOG region, we used inverse distance weighting (IDW) interpolation [39] to more accurately reflect the decrease in fetch during the transition from oceanic to coastal waters. For the NCC and SOG, offshore we calculated fetch points at a coarser resolution to support the interpolation, while for QCS, IDW was applied only to the high water line points since the region is largely sheltered and comprised of channels and inlets. |

*Table S3* *Cross-walk tables for the independent data sets used in this study. Translation of Canadian Hydrographic Service observations and Natural Resources Canada grab samples are described in [30].*

|  | **ROV** | **SCUBA** | **Drop camera** |
| --- | --- | --- | --- |
| Rock | Hardpan | Bedrock - smooth | Bedrock – smooth |
|  | Bedrock | Bedrock - crevices | Bedrock – crevices |
|  | Boulder^1^ | Boulder^1^ | Boulder^1^ |
| Mixed | Cobble^2^ | Cobble^2^ | Cobble^2^ |
|  | Mixed coarse^3^ | Gravel^3^ | Gravel^3^ |
|  | Gravel^4^ | Pea Gravel^4^ | Pea Gravel^4^ |
| Sand | Sand^5^ | Sand^5^ | Sand^5^ |
|  |  | Shell - crushed | Shell – crushed |
| Mud | Mud | Mud | Mud |
| Unused | Artificial | Not applicable | Not applicable |

Descriptions of substrate size from Davies et al. [43]:

1. Rocks larger than basketball

2. Rocks that are fist-sized to basketball-sized

3. Particles 1 cm to fist-sized

4. Particles 2 mm to 1 cm

5. Particles < 2 mm

Table S4: Error matrices for the six weighted models. Tables show the correspondence between model predictions (the rows) and the build testing partition (the reference data, in columns). Prevalence (Prev), showing the proportion of each class, and Producer (Prod) and User accuracies contribute to interpreting model performance.

***Coastwide***

|  | 1 | 2 | 3 | 4 | Prev | User |
| --- | --- | --- | --- | --- | --- | --- |
| 1 | 22616 | 2241 | 3825 | 1605 | 0.46 | 0.75 |
| 2 | 1431 | 3080 | 1290 | 318 | 0.09 | 0.50 |
| 3 | 2866 | 2076 | 12386 | 1151 | 0.28 | 0.67 |
| 4 | 1176 | 518 | 1164 | 8313 | 0.17 | 0.74 |
| Prev | 0.43 | 0.12 | 0.28 | 0.17 |  |  |
| Prod | 0.81 | 0.39 | 0.66 | 0.73 |  |  |

***HG***

|  | 1 | 2 | 3 | 4 | Prev | User |
| --- | --- | --- | --- | --- | --- | --- |
| 1 | 2451 | 304 | 418 | 81 | 0.35 | 0.75 |
| 2 | 150 | 467 | 202 | 24 | 0.09 | 0.55 |
| 3 | 317 | 555 | 2958 | 117 | 0.43 | 0.75 |
| 4 | 101 | 48 | 93 | 905 | 0.12 | 0.79 |
| Prev | 0.33 | 0.15 | 0.40 | 0.12 |  |  |
| Prod | 0.81 | 0.34 | 0.81 | 0.80 |  |  |

***NCC***

|  | 1 | 2 | 3 | 4 | Prev | User |
| --- | --- | --- | --- | --- | --- | --- |
| 1 | 95353 | 882 | 1513 | 378 | 0.55 | 0.77 |
| 2 | 243 | 652 | 229 | 60 | 0.05 | 0.55 |
| 3 | 1153 | 593 | 3499 | 284 | 0.25 | 0.63 |
| 4 | 391 | 171 | 338 | 2268 | 0.14 | 0.72 |
| Prev | 0.51 | 0.10 | 0.25 | 0.13 |  |  |
| Prod | 0.84 | 0.28 | 0.63 | 0.76 |  |  |

***WCVI***

|  | 1 | 2 | 3 | 4 | Prev | User |
| --- | --- | --- | --- | --- | --- | --- |
| 1 | 4569 | 326 | 370 | 159 | 0.57 | 0.84 |
| 2 | 227 | 635 | 195 | 65 | 0.12 | 0.57 |
| 3 | 240 | 209 | 1156 | 133 | 0.18 | 0.67 |
| 4 | 123 | 91 | 138 | 887 | 0.13 | 0.72 |
| Prev | 0.54 | 0.13 | 0.20 | 0.13 |  |  |
| Prod | 0.89 | 0.50 | 0.62 | 0.71 |  |  |

***QCS***

|  | 1 | 2 | 3 | 4 | Prev | User |
| --- | --- | --- | --- | --- | --- | --- |
| 1 | 1951 | 220 | 231 | 120 | 0.58 | 0.77 |
| 2 | 64 | 184 | 52 | 13 | 0.07 | 0.59 |
| 3 | 162 | 110 | 501 | 81 | 0.19 | 0.59 |
| 4 | 107 | 52 | 88 | 447 | 0.16 | 0.64 |
| Prev | 0.52 | 0.13 | 0.20 | 0.15 |  |  |
| Prod | 0.85 | 0.33 | 0.57 | 0.68 |  |  |

***SOG***

|  | 1 | 2 | 3 | 4 | Prev | User |
| --- | --- | --- | --- | --- | --- | --- |
| 1 | 4808 | 570 | 774 | 308 | 0.45 | 0.74 |
| 2 | 236 | 573 | 249 | 52 | 0.08 | 0.52 |
| 3 | 498 | 340 | 2119 | 323 | 0.23 | 0.65 |
| 4 | 293 | 133 | 440 | 2683 | 0.25 | 0.76 |
| Prev | 0.41 | 0.11 | 0.25 | 0.23 |  |  |
| Prod | 0.82 | 0.35 | 0.59 | 0.80 |  |  |


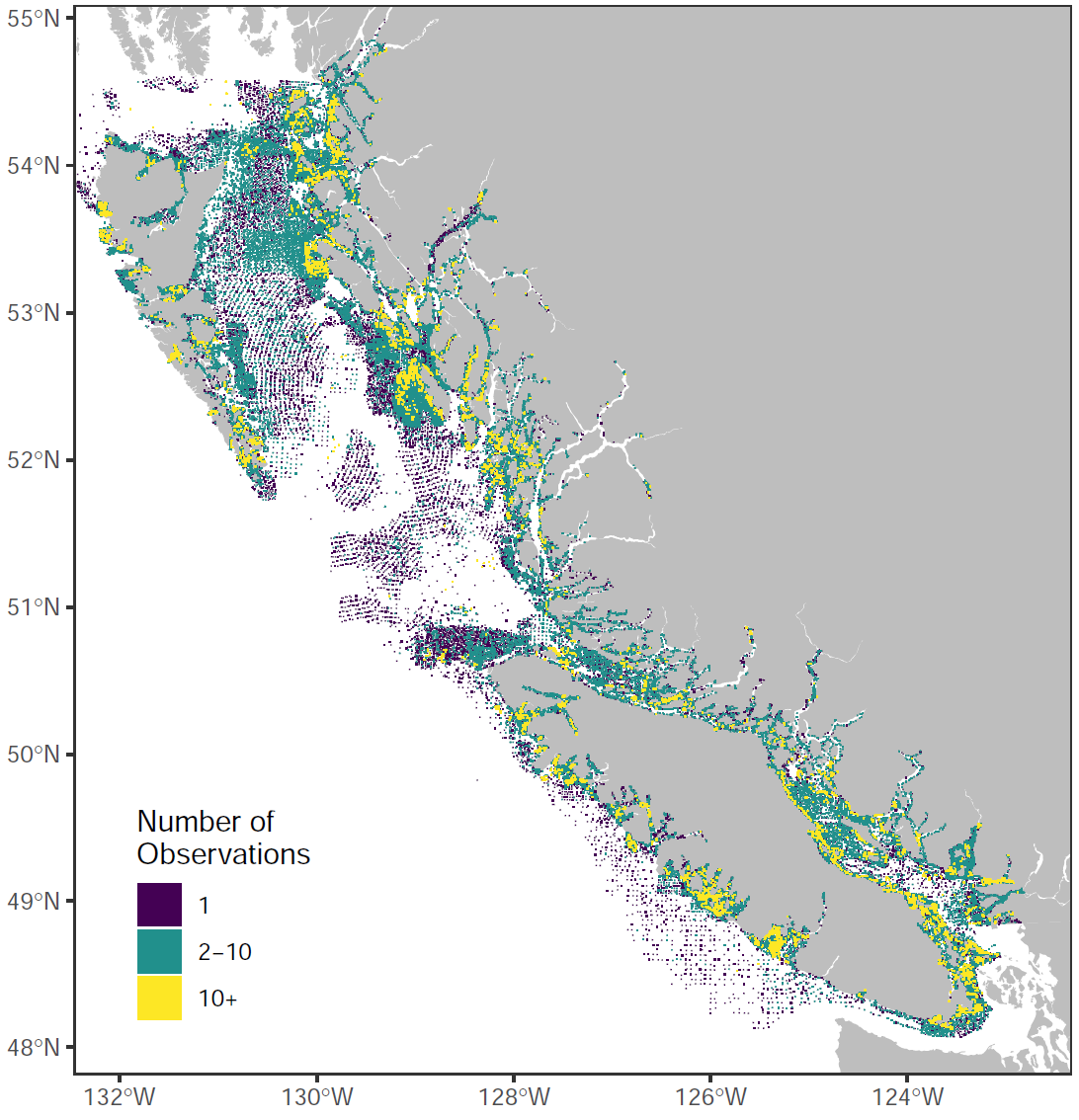


*Figure S1: Distribution of the 197,587 substrate observations used for model development (the Build data) binned to a grid of 1,000 x 1,000 m^2^ polygons. Multiple observations in a raster cell are the result of repeated surveys and the binning the data (see S1 Table) not pseudo-replication of transect data. We chose to treat these repeated observations as independent samples because they were unlikely to have been sampled the exact same location, and thereby provide important information to the model by either re-enforcing the association with the predictors if they agree, or by reflecting local heterogeneity if they differ.*


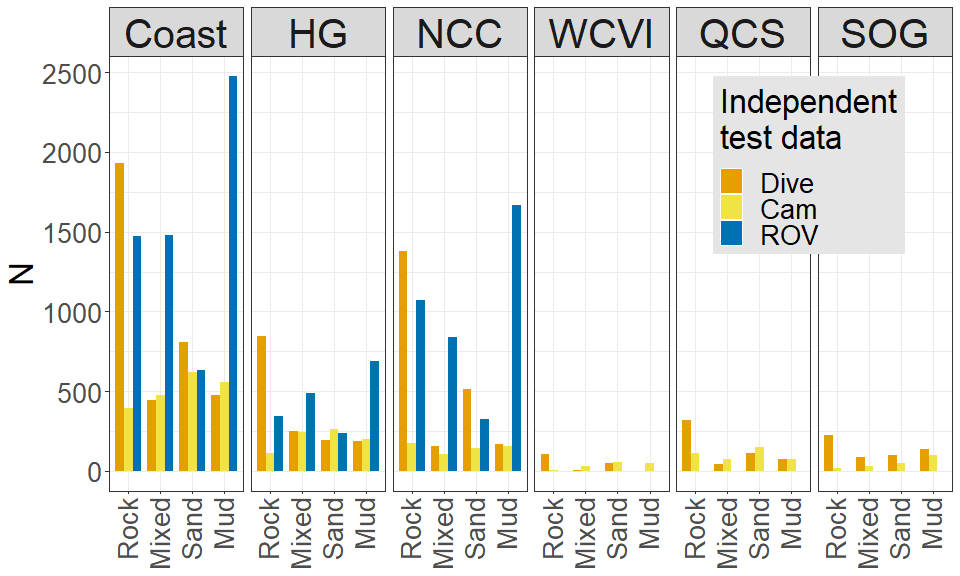


*Figure S2: Samples size of independent data sets by substrate class, across regions.*


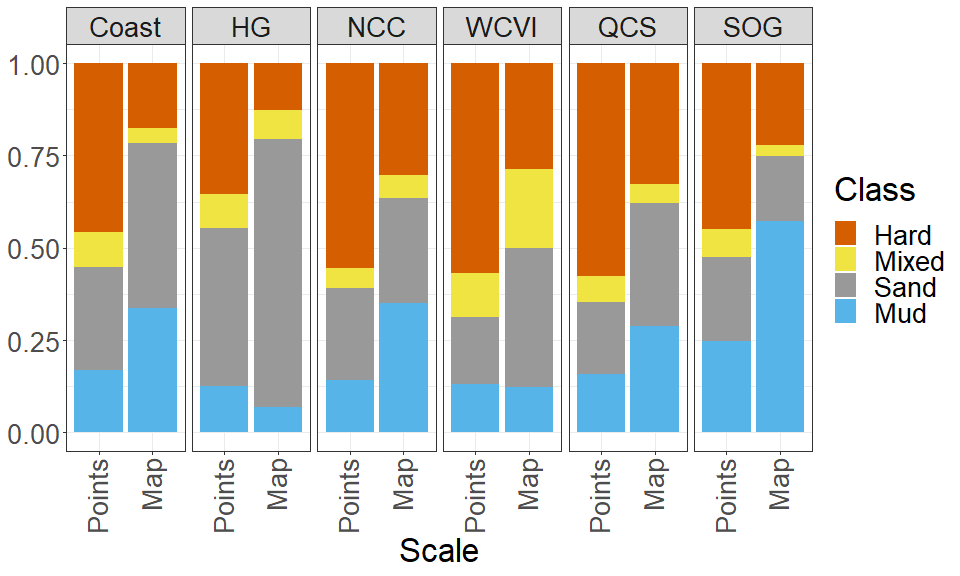


*Figure S3: Prevalence in models fitted to observations (Points) versus mapped predictions (Map).*

*
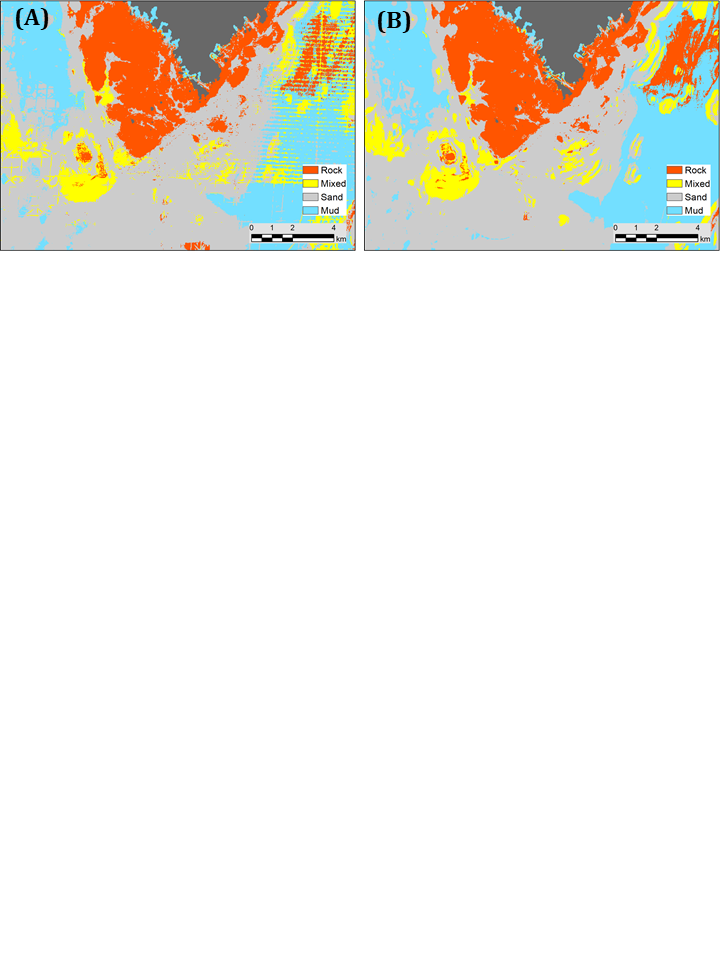
**Figure S4: Artefacts in the substrate predictions (A) and their reduction via smoothing of the bathymetry layer prior to generating bathymetric derivatives (B).*


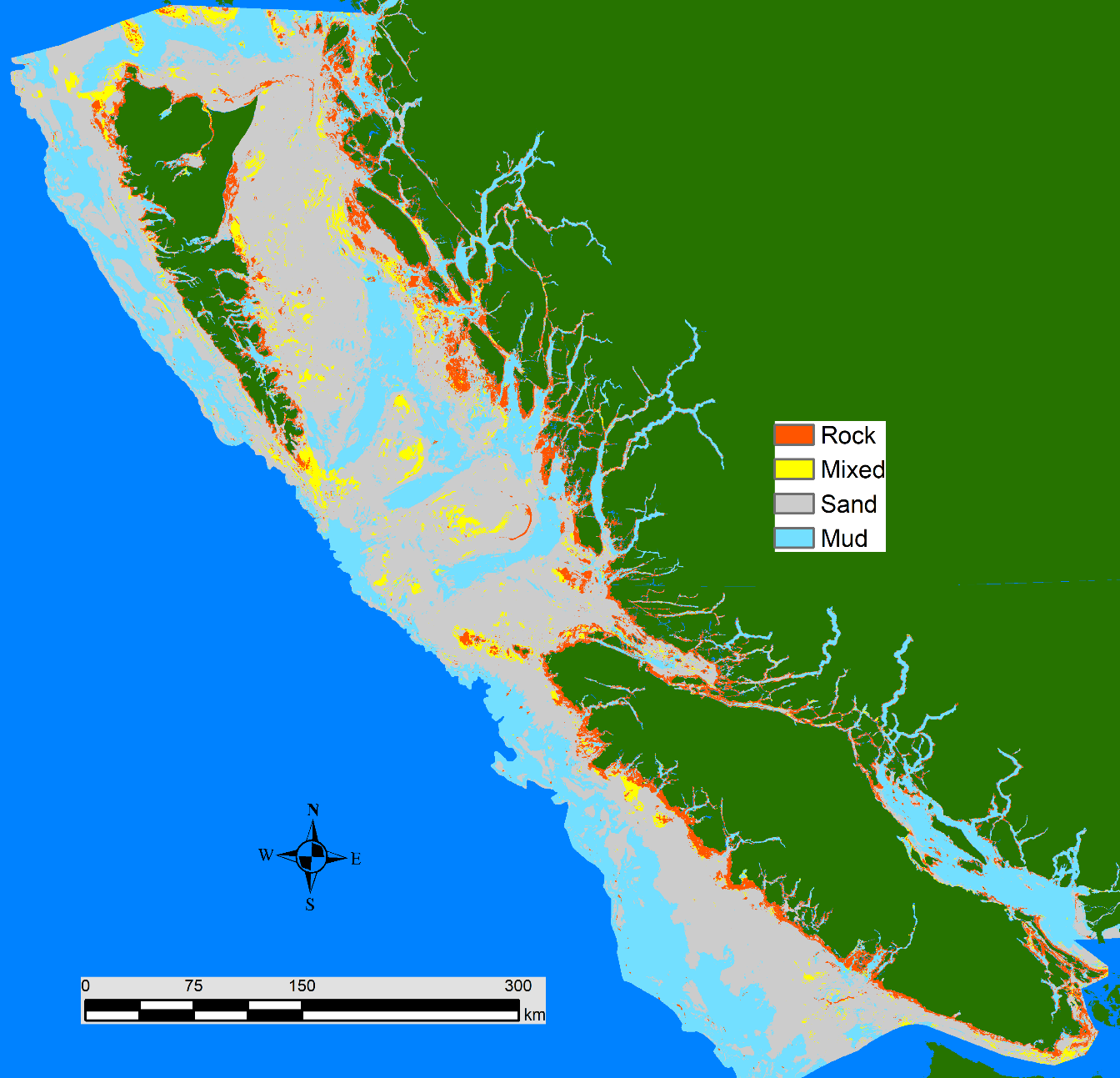
*Figure S5: Coastwide predictions of bottom type for Canada’s Pacific shelf. Predictive model of bottom type on a 100 x 100 m^2^ grid based on correlations between 197,587 observations of substrate and eight geomorphic and three energy predictors.*
