# Supplementary material for: Comprehensive marine substrate classification applied to Canada’s Pacific shelf
Source: PLoS One. 2021 Oct 29;16(10):e0259156. doi: 10.1371/journal.pone.0259156 (PMC8555849; doi:10.1371/journal.pone.0259156)
Supplement: Attachment — Submitted filename: PONE-D-20-40989_reviewer.pdf [file pone.0259156.s002.pdf]

# PLOS ONE

## Comprehensive marine substrate classification of Canada’s Pacific shelf

--Manuscript Draft--

| | |
|---|---|
| **Manuscript Number:** | PONE-D-20-40989 |
| **Article Type:** | Research Article |
| **Full Title:** | Comprehensive marine substrate classification of Canada’s Pacific shelf |
| **Short Title:** | Substrate of Canada’s Pacific shelf |
| **Corresponding Author:** | Edward J Gregr<br>University of British Columbia<br>Vancouver, British Columbia CANADA |
| **Keywords:** | substrate; random forest; bottom type; habitat; benthic; coastal; shelf |
| **Abstract:** | Maps of bottom type are essential to the management of marine resources and biodiversity because of their foundational role in characterizing species’ habitats. They are also urgently needed as countries work to define marine protected areas. Current approaches are time consuming, focus largely on grain size, and tend to overlook shallow waters. Our random forest classification of almost 200,000 observations of bottom type is a timely alternative, providing maps of coastal substrate at a combination of resolution and extents not previously achieved. We correlated the observations with depth, depth-derivatives, and estimates of energy to predict marine substrate at 100 m resolution for Canada’s Pacific shelf, a study area of over 135,000 km2. We built five regional models with the same data at 20 m resolution. In addition to standard tests of model fit, we used three independent data sets to test model predictions. We also tested for regional, depth, and resolution effects. We guided our analysis by asking: 1) does weighting for prevalence improve model predictions? 2) does model resolution influence model performance? And 3) is model performance influenced by depth? All our models fit the build data well with true skill statistic (TSS) scores ranging from 0.79 to 0.82. Weighting models with class prevalence improved fit and the correspondence with known spatial features. Class-based metrics showed differences across both resolutions and spatial regions, emphasizing the value of disaggregated metrics to understanding process stationarity. Predictive power was lower (TSS from 0.53 to 0.68) when models were evaluated with independent data sets, emphasizing how this is different from model fit. Model performance was a function of both depth and resolution, illustrating the challenge of accurately representing heterogeneity. Our work shows how independent data improves understanding of model performance and sampling bias, and the value of regional analyses to assessing model stationarity. |
| **Order of Authors:** | Edward J Gregr |
| | Dana Haggarty |
| | Sarah Davies |
| | Cole Fields |
| | Joanne Lessard |
| **Additional Information:** | |
| **Question** | **Response** |
| **Financial Disclosure**<br><br>Enter a financial disclosure statement that describes the sources of funding for the work included in this submission. Review the submission guidelines for detailed requirements. View published research articles from *PLOS ONE* for specific | This work was funded by the Government of Canada World Class Oil Spill Response initiative overseen by the Department of Fisheries and Oceans. |

| | |
|---|---|
| examples.<br><br>This statement is required for submission and **will appear in the published article** if the submission is accepted. Please make sure it is accurate.<br><br>**Unfunded studies**<br>Enter: *The author(s) received no specific funding for this work.*<br><br>**Funded studies**<br>Enter a statement with the following details:<br>• Initials of the authors who received each award<br>• Grant numbers awarded to each author<br>• The full name of each funder<br>• URL of each funder website<br>• Did the sponsors or funders play any role in the study design, data collection and analysis, decision to publish, or preparation of the manuscript?<br>• **NO** - Include this sentence at the end of your statement: *The funders had no role in study design, data collection and analysis, decision to publish, or preparation of the manuscript.*<br>• **YES** - Specify the role(s) played.<br><br>* typeset | |
| **Competing Interests**<br><br>Use the instructions below to enter a competing interest statement for this submission. On behalf of all authors, disclose any competing interests that could be perceived to bias this work—acknowledging all financial support and any other relevant financial or non-financial competing interests.<br><br>This statement **will appear in the published article** if the submission is accepted. Please make sure it is accurate. View published research articles from *PLOS ONE* for specific examples. | The authors declare no competing interests. |

| | |
|---|---|
| **NO authors have competing interests**<br><br>Enter: *The authors have declared that no competing interests exist.*<br><br>**Authors with competing interests**<br><br>Enter competing interest details beginning with this statement:<br><br>*I have read the journal's policy and the authors of this manuscript have the following competing interests: [insert competing interests here]*<br><br>* typeset | |
| **Ethics Statement**<br><br>Enter an ethics statement for this submission. This statement is required if the study involved:<br><br>• Human participants<br>• Human specimens or tissue<br>• Vertebrate animals or cephalopods<br>• Vertebrate embryos or tissues<br>• Field research<br><br>Write "N/A" if the submission does not require an ethics statement.<br><br>General guidance is provided below. Consult the submission guidelines for detailed instructions. **Make sure that all information entered here is included in the Methods section of the manuscript.** | No research was conducted on human or animal subjects. |

| | |
|---|---|
| **Format for specific study types**<br><br>**Human Subject Research (involving human participants and/or tissue)**<br>• Give the name of the institutional review board or ethics committee that approved the study<br>• Include the approval number and/or a statement indicating approval of this research<br>• Indicate the form of consent obtained (written/oral) or the reason that consent was not obtained (e.g. the data were analyzed anonymously)<br><br>**Animal Research (involving vertebrate animals, embryos or tissues)**<br>• Provide the name of the Institutional Animal Care and Use Committee (IACUC) or other relevant ethics board that reviewed the study protocol, and indicate whether they approved this research or granted a formal waiver of ethical approval<br>• Include an approval number if one was obtained<br>• If the study involved *non-human primates*, add *additional details* about animal welfare and steps taken to ameliorate suffering<br>• If anesthesia, euthanasia, or any kind of animal sacrifice is part of the study, include briefly which substances and/or methods were applied<br><br>**Field Research**<br><br>Include the following details if this study involves the collection of plant, animal, or other materials from a natural setting:<br>• Field permit number<br>• Name of the institution or relevant body that granted permission | |
| **Data Availability**<br><br>Authors are required to make all data underlying the findings described fully available, without restriction, and from the time of publication. PLOS allows rare exceptions to address legal and ethical concerns. See the PLOS Data Policy and FAQ for detailed information. | Yes - all data are fully available without restriction |

| | |
|---|---|
| A Data Availability Statement describing where the data can be found is required at submission. Your answers to this question constitute the Data Availability Statement and **will be published in the article**, if accepted.<br><br>**Important:** Stating 'data available on request from the author' is not sufficient. If your data are only available upon request, select 'No' for the first question and explain your exceptional situation in the text box.<br><br>Do the authors confirm that all data underlying the findings described in their manuscript are fully available without restriction? | |
| **Describe where the data may be found in full sentences. If you are copying our sample text, replace any instances of XXX with the appropriate details.**<br><br>• If the data are **held or will be held in a public repository**, include URLs, accession numbers or DOIs. If this information will only be available after acceptance, indicate this by ticking the box below. For example: *All XXX files are available from the XXX database (accession number(s) XXX, XXX.).*<br>• If the data are all contained **within the manuscript and/or Supporting Information files**, enter the following: *All relevant data are within the manuscript and its Supporting Information files.*<br>• If neither of these applies but you are able to provide **details of access elsewhere**, with or without limitations, please do so. For example:<br><br>*Data cannot be shared publicly because of [XXX]. Data are available from the XXX Institutional Data Access / Ethics Committee (contact via XXX) for researchers who meet the criteria for access to confidential data.*<br><br>*The data underlying the results presented in the study are available from (include the name of the third party* | The data underlying the results presented in the study will be available from GitHub. |

| | |
|---|---|
| *and contact information or URL).*<br>• This text is appropriate if the data are owned by a third party and authors do not have permission to share the data.<br><br>* typeset | |
| Additional data availability information: | Tick here if the URLs/accession numbers/DOIs will be available only after acceptance of the manuscript for publication so that we can ensure their inclusion before publication. |

Manuscript
Click here to access/download;Manuscript;substrate paper Text_3.2a_PLOS.docx

# Comprehensive marine substrate classification of Canada's Pacific shelf

Edward Gregr[1*], Dana Haggarty[2], Sarah Davies[2], Cole Fields[3], and Joanne Lessard[2]

1. SciTech Environmental Consulting, 2136 Napier Street, Vancouver, British Columbia, V5L 2N9, Canada

2. Fisheries and Oceans Canada, Pacific Biological Station, 3190 Hammond Bay Rd, Nanaimo, British Columbia, V9T 6N7, Canada

3. Fisheries and Oceans Canada, Institute of Ocean Sciences, 9869 W Saanich Rd, Sidney, British Columbia, V8L 5T5, Canada

* Corresponding Author:

Email: ed@scitechconsulting.com

# Abstract

Maps of bottom type are essential to the management of marine resources and biodiversity because of their foundational role in characterizing species' habitats. They are also urgently needed as countries work to define marine protected areas. Current approaches are time consuming, focus largely on grain size, and tend to overlook shallow waters. Our random forest classification of almost 200,000 observations of bottom type is a timely alternative, providing maps of coastal substrate at a combination of resolution and extents not previously achieved. We correlated the observations with depth, depth-derivatives, and estimates of energy to predict marine substrate at 100 m resolution for Canada's Pacific shelf, a study area of over 135,000 km$^2$. We built five regional models with the same data at 20 m resolution. In addition to standard tests of model fit, we used three independent data sets to test model predictions. We also tested for regional, depth, and resolution effects. We guided our analysis by asking: 1) does weighting for prevalence improve model predictions? 2) does model resolution influence model performance? And 3) is model performance influenced by depth? All our models fit the build data well with true skill statistic (TSS) scores ranging from 0.79 to 0.82. Weighting models with class prevalence improved fit and the correspondence with known spatial features. Class-based metrics showed differences across both resolutions and spatial regions, emphasizing the value of disaggregated metrics to understanding process stationarity. Predictive power was lower (TSS from 0.53 to 0.68) when models were evaluated with independent data sets, emphasizing how this is different from model fit. Model performance was a function of both depth and resolution, illustrating the challenge of accurately representing heterogeneity. Our work shows how independent data improves understanding of model performance and sampling bias, and the value of regional analyses to assessing model stationarity.

# Introduction

Understanding how marine species are distributed is central to ecosystem-based fisheries management, vulnerability assessments, protected area design, and other marine spatial planning activities such as aquaculture siting and oil spill response. This understanding relies on models of habitat suitability [e.g., 1, 2], the credibility of which depends in large part on the accuracy of the underlying environmental predictors. While often described as habitat in the marine classification literature [e.g., 3], substrate is more accurately described as a key determinant of habitat for benthic species [4, 5]. However, unlike predictors of ocean dynamics or chemistry (now regularly obtained via remote sensing or ocean circulation models), reliable descriptions of bottom type continue to elude researchers due to both sampling and analytic challenges. As countries continue to increase their protection of marine spaces [6, 7], effective management will require this knowledge gap to be filled efficiently and quantitatively [8].

In this contribution, we used a random forest classification to develop a substrate model for Pacific Canadian shelf waters at two spatial resolutions (20 m and 100 m). We extended the rocky reef model of Haggarty and Yamanaka (201[illegible]) to include Mixed, Sand, and Mud substrates, and added energy predictors (i.e., wave fetch, bottom current, and tides) and additional bathymetric derivatives. Our methods move beyond random forest regression models of acoustically collected data [e.g., 9, 10] by mobilizing available observations of bottom type from multiple sources. We evaluated model performance (both model fit and predictive power) across resolutions, geographic regions, and depths. We present our results using a collection of diverse and interpretable metrics.

We found model performance differed across regions and depths, and that this was mediated by model resolution. Our analysis also showed the importance of weighting for prevalence, diverse evaluation metrics, and qualitative assessments of mapped predictions.

# Background

The emergence of multibeam (MB) echosounder acoustics in the early 2000s [11] revolutionized how oceanographers and marine geologists view the sea floor. Prior to MB swath scanners, sea floor characteristics (depth and substrate) were inferred from point observations. Today, in addition to sub-meter bathymetric models, seabed sediment classifications can be derived from the acoustic backscatter (BS) intensity [e.g., 12] often collected simultaneously with the MB bathymetry. The strength of the BS signal reflects the hardness of the bottom, with strong signals typically representing hard, and weak signals representing soft substrates [13].

The allure of acoustic data to estimate sediment composition is strong because swath sensors can cover large areas at high resolution, and are increasingly efficient with depth. However, comprehensive acoustic surveys that include shallow coastal areas are expensive and time consuming, particularly for less developed countries and complex coastlines, and can take decades to completely map [14]. Further, the value of developing BS-based classifications is unclear: available evidence suggests including BS data provides little improvement in some contexts [see 15, 16]; and a recent review BS classification methods identified ongoing challenges relating to standardized signal calibration, data rectification, and ground-truthing [17].

In British Columbia, Canada, only a portion of the continental shelf has been mapped with MB, and MB echosounder data are particularly lacking in the complex, intertidal and shallow waters (Cooper Stacey, personal communication). Further, because Individual MB surveys are typically acquired with different parameters, it is difficult to automate an analysis between surveys, making the process extremely labor and time intensive [17]. It will therefore not be possible to create a comprehensive MB BS substrate map for the Canadian Pacific coast for decades.

## Objectives

Our main objective was to build a comprehensive, ecologically-relevant coastwide map of marine substrate. Since protecting quality habitat can support both conservation and fisheries objectives [18], building an understanding of species' habitats is central to marine spatial planning. This makes timeliness an additional objective, and necessitates using the best available data. We approached this objective by building models extending from the high water line to the continental shelf. We defined classes to capture all substrate types (although with low class resolution), making them more relevant than grain size or single substrate models. Our methods are transparent and reproducible allowing refinement and updates as required - an advantage given ongoing data collection [14].

To assess the reliability of our model, we examined how prevalence, sampling bias, model resolution, and depth interact to influence model performance. Specifically, we asked the following questions:

1) Can weighting classes by observation prevalence improve model predictions? While prevalence is known to affect random forest performance, this has not been quantitatively assessed in the substrate literature.
2) What are the effects of model resolution? We compared the performance of the regional and coastwide models, and used distinct geographic regions to examine model stationarity [i.e., their consistent parameterization over space or time - 19].
3) Do our models perform differently by depth? And if so, are these differences correlated with model resolution? Our collective experience, based on over 50 years of conducting surveys on the BC Coast, suggests that substrate heterogeneity (spatial variability) decreases with depth. We therefore predicted that higher resolution models would perform better in shallower waters.

# Challenges

## Ecological relevance

Given the role of substrate as a predictor for habitat suitability models and an indicator of environmental sensitivity, the utility of any substrate model lies in its ecological relevance. Two important aspects of relevance are the types of classes used, and the resolution of the analysis.

While many substrate classification papers signal their utility for defining habitat for species, most are ‘ecology free’, simply assuming the derived classes are ecologically relevant. For example, BS classification largely predicts particle size [20-22] to describe the soft sediment component of the European nature information system (EUNIS) marine habitat classification. The EUNIS has a separate classification for rocky habitat, but classifications that integrate hard and soft substrates [e.g., 14, 21, 23] are less common in the literature. Any classification that excludes hard substrate will have limited ecological relevance, as it will not support models of epiphaunal organisms [e.g., 24] or those associated with high relief areas [e.g., 15]. Fortunately, while a representation of all bottom types is necessary to maximize relevance, the number of classes need not be large, since habitat models and ecological analyses typically don’t require and often cannot accommodate detailed classifications [e.g., 25]. We therefore limited our classification to four ecologically distinct classes: Rock, Mixed, Sand, and Mud. This has the advantage of allowing multiple sources of substrate observations to be combined[e.g., 14], and facilitates reproducibility compared to methods more closely tied to particular data types.

We recognize that for disciplines (e.g., marine geology) where the objective is to identify the range of geologically distinguishable bottom types, this coarse classification would be limiting. However, producing a classification that is both comprehensive and detailed is a big task, particularly since an increase in the number of classes tends to decrease model fit [22, 26]. For example, while Vasques et al. [27] identified 22 shallow and 25 deep EUNIS classes, the work

was augmented with expert interpretation. In our case, a focus on ecological relevance helped limit the number and types of classes required.

Our classification also needed to be comprehensive across space - from the high water line to the shelf break - to ensure broad relevance. The exclusion of the coastal zone [commonly called the white strip because of the absence of data - 14] is a chronic problem despite it being both the most productive region of the ocean and the most impacted by human activities [28, 29]. We addressed this challenge by using observations from across the entire range of depths from the intertidal to the shelf edge.

## Resolution

Developing relevant substrate maps is also challenged by the fractal nature of natural systems. This means that mapped substrate heterogeneity depends on the resolution used. For example, a 100 m x 100 m model assumes homogeneity across each pixel and, as the unit of analysis, point observations are aggregated to this resolution. This means the analysis can only represent variability at this single resolution. Matching the scale of analysis to the features of interest is therefore essential if it is to be accurately represented. Aggregating observations to a specific resolution also likely reduces the class accuracy. This suggests that when point observations are used for validation, model performance will increase with study resolution. This assertion is supported by contrasting the performance of recent random forest grain size models built at different resolutions [10, 26, 30], and suggests that model accuracy depends on sample distribution and density. We engage the challenges of resolution by comparing a model built at 100 m resolution to models built at 20 m resolution, the resolution of the available predictors. This is currently the finest resolution achievable across the large spatial extents of our study area. Compared to the most extensive comparable work recently published [10], our 100 m

study area is about 1/5th the size but with 25x better resolution, giving a data footprint (75 million pixels) 5 times larger. Our 20 m regional models range from 91 to 470 million pixels.

## Sampling

As implied above, how observations are collected, prepared, and structured can influence model performance. This sampling context [sensu 31] informs the types of models that can be developed (for example, our use of substrate observations from diverse sources precludes an analysis of grain size, and our use of a categorical dependent variable precludes the use of regression).

Part of the context of bottom type samples is they tend to be spatially patchy, and biased towards different bottom types and depths according to sampling method. For example, Lawrence et al. [8] describe the challenge of observational sampling when hard substrates are covered with a veneer of soft sediment. Thus, all data likely contain some error, either in the attribute measured, or its position. If such errors are systematic, they can lead to artefacts in the predictor layer. Examples include edge-effects in bathymetric derivatives generated by excluding land-side elevations [15, 32], and errors caused by the interpolation of unevenly sampled data (e.g., bathymetric track lines).

The degree of contextual overlap between data sets, which determines their shared biases, is particularly salient when testing predictive power. Understanding how the biases in test data influences model predictive power can provide insight into the limits of model complexity and improve understanding of model performance and scaling [24].

## Model development

Selecting the most suitable approach to relate observations and predictors given model objectives and available data is the crux of model development, which also includes the selection

of predictors, resolution, and performance metrics. For acoustic data collected with remote sensors, tree-based classifiers such as random forest are now the most common method applied [e.g., 20, 21]. Similar approaches are used in ecological studies to classify species observations into predictions of suitable habitat. In both cases, environmental predictors are used to support the classification. In habitat studies, the predictors are assumed to have a functional relationship with the observations, allowing the resulting models to interpolate habitat suitability predictions across the range of the predictors. The habitat suitability models therefore rely on the spatial auto-correlation of the predictors to make continuous habitat maps [19].

A fundamental difference between these two applications is that remote sensing data are (mostly) spatially comprehensive, so fitting the data is the principal goal - there is typically no need for a predictive component. Whereas a habitat map derived from species observations relies on the strength and stationarity of the predicted relationships. This is why tests of predictive power (see following section) are essential to evaluating point-based classifications used in habitat suitability modelling, and adapted here.

## Model performance

Evaluating model performance requires appropriate metrics, quality ground truthing or testing data, and in the case of point-based models, the consideration of process stationarity. Performance metrics have evolved little in over twenty years of predictive modelling [33], with most studies continuing to report Cohen’s Kappa as a measure of model quality, despite its shortcomings being well described for almost a decade [34, 35]. While alternatives continue to appear in the literature, progress in this regard has been glacial, at least in part because papers with equations tend to be poorly cited by many practitioners [36].

There is also a persistent misconception about how to interpret model performance, given the testing data. The situation is well described by Zhang et al. [37] who emphasize that correlations

between dependent and explanatory variables are likely to be reliable in the context of the training data, but reduced for other data contexts. The majority of models are tested using cross-validation (the splitting of a set of observations into training and testing partitions), and are therefore correctly described as internal validation [38] or tests of model fit. To test model performance outside the context of the training data, is it now generally acknowledged that independently collected data (i.e., data with other contexts) are needed [39-41]. In the ecological literature, such independent data evaluation (IDE) has been called external evaluation [38], tests of model transfer [41], and forecast skill [24]. We use the term predictive power [33] for its generality and clarity of purpose. While the utility of IDE has been principally assessed using data collected for purpose [40], the use of opportunistic data can serve a similar purpose, while also illustrating important differences in data context [e.g., 24].

Evaluating the role of space in models that map what are commonly aspatial correlative relationships [19] is also a significant challenge. Examining residuals is an important aspect of assessing spatial autocorrelation of continuous predictions and model stationarity [a common but generally false assumption - 24, 42], but we found no guidance on using residuals to evaluate categorical predictions. Other work has examined the spatial variability of model error by calculating performance metrics at a number of small, randomly positioned sites [43]. This approach was not feasible for our study because we could not filter for the effect of sampling density on model performance. We approached this challenge by testing for model stationarity across different regions and depths.

# Methods

We apply a random forest classification [44-46] to a collection of substrate observations to create predictive models of four substrate classes (Rock, Mixed, Sand, and Mud) based on a suite of geophysical predictors. Random forest is an attractive machine learning approach because of its

reliable performance [e.g., 20], reported insensitivity to overfitting the data [44, 47], and ability to accommodate a variety of relationships between observations and predictors [44, 45].

We built a 100 m (100 x 100 $m^2$) coastwide model for the Canadian Pacific continental shelf. To improve the quality of habitat models for species close to shore and extend the predictions across the white strip, we built 5 nested regional, nearshore models at 20 m resolution (20 x 20 $m^2$) (Fig. 1). We also built paired models to test for the effect of class prevalence. This collection of models allowed us to examine the relative performance of models across resolutions, regions, and depths.

**Fig 1. Study Area.** Map of study area showing outline of the six models developed in this analysis. The 100 m coastwide model covers the Canadian Pacific continental shelf. The five 20 m regional models extend from the high water line to as far as 5 km seaward from the 50 m depth contour, the limits of the bathymetry and derived predictor variables. See text for details.

We tested model fit by partitioning the data used to build the models (the build data, Table 1) into training and testing partitions. We tested the predictive power of our models using three independent data sets, collected separately. Our predictor variables (Table 2) included a commonly used suite of geomorphic predictors derived from bathymetry, and several measures of energy. We evaluated model performance using a suite of comprehensive and interpretable metrics. All analyses were done in R [48], using a number of packages for the data analysis and presentation of results (see S2 Analytical Methods).

**Table 1: Contents of the build data set showing number of observations (total and by substrate class) available for model development (Build) and independent model evaluation. Data types included Grab and Dive samples, observations from drop Cameras and remotely operated vehicles (ROV), and chart annotations (of Marsh). Data were sourced from the Canadian Hydrographic Service (CHS), Natural Resources Canada (NRCan), and Fisheries and Oceans Canada (DFO).**

| **Role** | **Type** | **Source** | **N** | **Class** | | | |
|---|---|---|---|---|---|---|---|
| | | | | *Rock* | *Mixed* | *Sand* | *Mud* |
| **Build** | Grab | CHS | 127,770 | 58,899 | 13,688 | 34,753 | 20,430 |
| | Grab | NRCan | 8,938 | 0 | 0 | 4,241 | 4,697 |
| | Dive | DFO | 44,809 | 21,250 | 7,626 | 13,464 | 2,469 |
| | ROV | DFO | 10,856 | 4073 | 2205 | 3200 | 1378 |
| | Marsh | CHS | 5,214 | 0 | 0 | 0 | 5,214 |
| | **Totals** | | 197,587 | 84,222 | 23,519 | 55,658 | 34,188 |
| **Evaluation** | Dive | DFO | 4974 | 2,892 | 543 | 974 | 565 |
| | Camera | DFO | 2143 | 421 | 491 | 654 | 577 |
| | ROV | DFO | 6064 | 1,477 | 1,479 | 633 | 2,475 |
| | **Totals** | | 13,181 | 4790 | 2513 | 2261 | 3617 |

**Table 2: Predictors used to classify the observational data and the data sources from which they were derived.**

| Predictor layer | Source | Native resolution | Study area | Number of Layers |
|---|---|---|---|---|
| Depth<br>Slope<br>Slope (std dev)<br>Curvature<br>Rugosity<br>Broad BPI<br>Medium BPI<br>Fine BPI | Carignan et al. (2013) elevation model. | 3 arc-seconds (~90 x 90 $m^2$) | Coastwide | 16[1] |
| | Gregr (2012) elevation model | 100 x 100 $m^2$ | | |
| | Davies et al. (2019) elevation models. | 20 x 20 $m^2$ | Regional | |
| Tidal speed<br>Ocean circulation | Mean summer conditions averaged from a regional circulation model (Masson and Fine 2012) | 3 x 3 $km^2$ | Coastwide<br>Regional | 2 |
| | Mean summer conditions averaged from a local circulation model (Soontiens et al. 2016). | 440 x 500 $m^2$ | Regional (SOG) | 2 |
| Fetch | Sum of fetch based on Gregr (2014) | 50 m | Regional | 1 |

All observational and predictor data were prepared using ArcGIS [49] before importing into R, where we joined all observational data with predictors scaled to coastwide (100 x 100 $m^2$) and regional (20 x 20 $m^2$) grids (i.e., rasters) before analysis. Predictors were not transformed or assessed for correlation since the random forest approach is largely robust to non-normal, correlated predictors [44, 45]. Where multiple observations occurred in the same raster cell, the predictor values were duplicated to preserve the observational sample size. This was more common for the coastwide model.

Models were built using the ranger package [50]. While several random forest packages are available, we found ranger was the only one to effectively support the weighting of classes based on prevalence. This is critical because the challenges posed by imbalanced datasets to machine learning methods is significant [51]. Earlier work [15] using similar data compared a well-balanced binary classification of rocky reefs (Rock, Not Rock) with the imbalanced classes used here and showed the greedy nature of the random forest algorithm towards an over-sampled class [51]. This led us to explore approaches to addressing imbalance. We found that weighting classes according to prevalence afforded a similar benefit to a 2-step procedure where the dominant class, and the three more balanced ones are classified separately and then combined [see 9].

We used the same number of trees (1000) and test fraction (0.6) for all ranger models. We set variable importance to use the Gini index for classification, and the internal cross-validation to sample with replacement. We applied the standard data partitioning approach of dividing the build data into training (67%) and testing (33%) partitions. We used the same training and testing partitions to build all the models, assess model fit, and explore the effects of depth and model resolution. Models were further assessed using IDE to test predictive power, examine sample bias, and test for stationarity.

## Addressing our objectives

The effect of class prevalence on habitat models has been known for some time [e.g., 52, 53]. It is also a significant area of research in the machine learning community [51], where well-balanced classes are encouraged. Finding little on this topic in the marine substrate classification literature, we tested for this effect by constructing a parallel set of models with and without class-size weighting. We weighted classes according to their prevalence in the training data, and compared their performance using both the build testing sample and the independent data sets. To assess if different classes were more reliably predicted with class weighting, we compared class-based statistics across all models. We also considered whether any observed differences were influenced by model resolution, as well as by region and depth.

It is reasonable to expect that physical and ecological processes will differ across regions with different spatial contexts. However, the detection and interpretation of this non-stationarity is rarely done. We tested whether our models performed better in some regions than others, and whether this was affected by model resolution. In particular, we were interested in whether a single coastwide model would perform similarly across large, physiographically distinct regions, and whether model resolution influenced performance across depths. This type of spatial analysis lends itself to tests for ecologically relevant spatial structure [40], and we use it here to assess how model performance might differ across space.

We compared model performance across regions and depth classes. To test for stationarity at the regional scale, we examined how the coastwide model performed in each of five regions (Fig. 1). These ranged from the sheltered, largely muddy Strait of Georgia (SOG) dominated by siltation from the Fraser River, to the exposed West Coast of Vancouver Island (WCVI), and the oceanographically distinct islands of Haida Gwaii (HG). The North Central Coast (NCC) includes deep fjords and inlets as well as a large exposed coastline, while the transitional Queen

Charlotte Strait (QCS) region includes sheltered and exposed areas, fjords and inlets. The many fjords are effective sinks for fine sediment and limit its distribution across the shelf [54].

We tested our hypothesis that model performance is correlated with depth by comparing model fit and predictive performance across a range of depth classes. We classified depths following Gregr et al. [14], who provided an ecological rationale for dividing coastal waters into Intertidal, 0 - 5 m, 5 - 10 m, 10 - 20 m, and 20 - 50 m zones. To these we added three deeper zones (50 - 100 m, 100 - 200 m, and 200+ m) for a more complete, shelf-wide comparison of depths.

## Model evaluation

We first assessed how well the models explain the testing partition of the build data (i.e., the model fit), and compared the weighted and non-weighted models to illustrate the effects of class prevalence. We used the same training and testing data partitions to test the fit of all models to standardize model comparison: because the testing partition has the same context as the training data, it can be considered unbiased and fully stationary test sample. These results thus provided a baseline for assessing the effects of sampling bias during the subsequent IDE to assess model predictive power. IDE is recognized as the gold standard for evaluating model predictive power because it tests how well models perform outside the context of the data used to build them [39].

To ensure we used the most informative performance statistics, we explored a variety of metrics developed across the disciplines of image classification [55, 56] habitat suitability modelling [33, 38], and weather forecasting [57]. To make our assessments comprehensive, we adapted complimentary metrics from across these different disciplines.

Best practice for assessing model performance calls for using more than one metric [39]. We report metrics describing both model accuracy and model error. Accuracy can measure either how much better model predictions are than a random guess, or the observed agreement

between predictions and a test dataset. For better-than-random, we use the True Skill Statistic (TSS) instead of Kappa because the TSS is corrected for random guessing and Kappa is known to be sensitive to prevalence [e.g., 52] and has limited utility as a performance metric [34, 35]. We use Normalized Accuracy and True Negative Rate (TNR) to provide information on false positives and false negatives. These metrics of better-than-random performance are relative to a no-information baseline (see Supplementary Methods S1).

We also report the Accuracy and the True Negative Rate (TNR) as measures of correct predictions of presence and absences, respectively. Measures of model error are based on the work of Pontius and colleagues [e.g., 56] and focus on understanding the nature of the errors in the classification. These include Quantity error, which is related to prevalence, Exchange error defined as a swapping between two categories, and Shift error, the remaining error that cannot be attributed to either Exchange or Quantity. These three error metrics are related to overall Accuracy in a straight-forward way:

$$\text{Accuracy} = 1 - \text{Quantity} - \text{Exchange} - \text{Shift}$$

We report these metrics both in aggregate (combined across classes), and at a class-based level. This combination of accuracy and error assessment provides a more complete picture of model performance than is commonly provided in such analyses. Finally, we offer Imbalance as an integrated measure of prevalence in a multi-class data set. See Supplementary Methods S1 for additional details.

To complement the quantitative assessment, we examined the spatial agreement of our predictions with two well-known areas of the Pacific coast, Pacific Rim National Park Reserve in the WCVI region, and English Bay, part of the urban coast of Greater Vancouver in the SOG region. This qualitative comparison adds valuable spatial context to the assessment of the substrate predictions by allowing the patterns produced by different models to be compared.

## Model build data

We assembled a coast-wide data set of 197,587 observations (Table 1) from Natural Resources Canada (NRCan), the Canadian Hydrographic Service (CHS) and Fisheries and Oceans Canada (DFO) data holdings to build the models. CHS collects grab samples as part of their regular hydrographic surveys. The observations are distributed across Pacific Canada, and thus represent the largest component of our build data set. However, sampling is biased towards shallow waters and hard substrate because of the CHS's mandate to chart navigable waterways. We therefore included NRCan grab data and marsh locations mapped by CHS to increase the prevalence of the soft bottom type classes in our build data. NRCan core samples are biased towards unconsolidated substrates. Direct observations of bottom type were acquired from DFO shellfish dive surveys and remotely operated vehicle (ROV) surveys of rockfish habitat. Observations were cross-walked to the four bottom type classes used in this analysis following Gregr et al. (2013). Details on these data are provided in Table S1.

## Predictor data

Environmental predictors were selected to include a combination of benthic terrain features derived from bathymetry [e.g., 15, 58] and measures of energy [e.g., 2, 59]. To support our analysis across two spatial resolutions, we derived the same terrain features from a 100 m coastwide bathymetry and our 20 m regional bathymetries. These included slope, curvature, rugosity, the standard deviation of slope, and three bathymetric positioning indices (BPIs) with increasing neighborhood sizes (Table S2) to capture both small benthic features, and larger trends in terrain. Energy was represented using tidal currents and broad-scale circulations derived from ocean current models [60, 61]. Fetch, a proxy for wind-wave exposure [62], was included in the 20 m model but not the 100 m model as the shallowest accurate prediction from the 100 m was expected to be deeper than shoaling depth. Additional details on the derivation of

these predictors is provided in Table S2. We did not explore questions of predictor independence or variable selection.

The 20 m bathymetry models we used extend 5 km inland to allow changes in elevations throughout the intertidal region to be characterized accurately. Terrestrial data ensured the interpolation across the high water line accurately captured coastal features such as estuaries and other low-lying areas as well as in steep-sided fjords (Davies et al. 2019). We ran the 20 m bathymetric derivatives with the terrestrial elevations in order to avoid edge effects in our predictors.

## Independent evaluation data

We sourced independent data from three sets of surveys with different passive observation techniques: dive surveys, drop cameras, and remotely operated video (ROV). All three datasets had consistently coded substrate classes making it easy to reclassify them to our four substrate classes (Table S3). All data were collected on transects. For this analysis, we aggregated the observations from each transect by 20 m grid cell and assigned the mode substrate observation to the center point of the cell.

Substrate observations from dive data (independent of the build data set) were collected from 1077 DFO dive transects surveyed across the coast between 2013 and 2018 to support the development of the West Coast Tanker Safety System. Data were collected following Davies et. al. [32] in shallow areas along the coast, ranging from -5 to 19 m depth. Points above chart datum were surveyed by divers at a high tide. Data were prepared in the same way as the dive component of the Build data (Table S1).

Drop camera observations were collected [following 32] using a GoPro camera deployed off the side of small boats during the DFO dive surveys (2014 to 2018). We obtained still photos from 889 locations at depths from 16 to 60 m. These data extended the dive observations of substrate

into deeper waters. Uncertainty in the positional accuracy of the images increases with depth due to deflection of the drop camera from the boat position. We minimized this uncertainty by removing locations where the difference between recorded depth and bathymetry exceeded 50 m.

ROV observations for Haida Gwaii and the North Central Coast regions were extracted from video imagery along 366 transects in depths from 33 to 675 m collected by DFO between 2013 and 2015. Observations were recorded for 10 second increments of video and aggregated into 20 m bins as described above.

# Results

Our results show how class weighting to address sample imbalance can lead to both numerical and spatial improvements in model performance. We also confirm the existence of regional non-stationarity, and show that model performance is correlated with depth. The results are presented in detail in the following sections.

## Model development and class weighting

All models, weighted or not, had comparable and high fit to data (TSS values ranging from 0.78 to 0.82) with no notable differences between the coastwide and the regional models in the aggregated metrics (Table 3). Accuracy was consistently lower than TNR; however, this is expected given the different, no-information baselines. The benefits of weighting for prevalence begin to emerge when the class-based metrics are examined (Fig. 2). Comparing the effect of weighting using class-based metrics (Fig. 2) showed Accuracy shifting away from Rock to the other classes, particularly Mixed and Mud, for all models. There was a corresponding increase in the Reliability score of the Rock class at the expense of the other classes, though this was more variable across models. The TNR changed less than Accuracy in response to weighting,

contradicting the observation by Allouche et al. [52] that prevalence has a greater influence on TNR than Accuracy. Across classes, Rock and Mud had the highest Accuracy and Reliability scores and the Mixed class had the lowest across all models, regardless of weighting (Fig. 2), confirming that this class gives the models the most difficulty. However, its higher TNR shows it was rarely predicted in error, perhaps in part due to its relatively small sample size (Fig. 3, Table S3).

**Table 3: Aggregated build metrics for all 6 models comparing the weighted (first row) and the unweighted (second row) random forest results. Out of bag (OOB) values show the mean prediction error from the random forest internal re-sampling. The true skill statistic (TSS) corrects Accuracy for chance and prevalence expressing how model performance exceeds random, while Accuracy, Specificity, Exchange and Shift provide an assessment of model error. See Supplementary materials S1 for more details on the metrics.**

| Model | N | Imbalance | OOB | TSS | Accuracy | TNR | Quantity | Exchange | Shift |
|---|---|---|---|---|---|---|---|---|---|
| Coast | 66056 | 0.17 | 0.30 | 0.79 | 0.70 | 0.86 | 0.03 | 0.25 | 0.02 |
| | | | *0.29* | *0.79* | *0.70* | *0.85* | *0.07* | *0.20* | *0.02* |
| HG | 9191 | 0.17 | 0.26 | 0.82 | 0.74 | 0.88 | 0.06 | 0.19 | 0.02 |
| | | | *0.25* | *0.82* | *0.74* | *0.87* | *0.08* | *0.17* | *0.01* |
| NCC | 22189 | 0.22 | 0.28 | 0.79 | 0.72 | 0.83 | 0.05 | 0.21 | 0.02 |
| | | | *0.28* | *0.79* | *0.72* | *0.80* | *0.10* | *0.16* | *0.02* |
| QCS | 4383 | 0.20 | 0.29 | 0.78 | 0.70 | 0.82 | 0.06 | 0.22 | 0.02 |
| | | | *0.29* | *0.79* | *0.71* | *0.79* | *0.11* | *0.16* | *0.02* |
| SOG | 14399 | 0.15 | 0.30 | 0.80 | 0.71 | 0.87 | 0.06 | 0.23 | 0.01 |
| | | | *0.30* | *0.80* | *0.71* | *0.86* | *0.08* | *0.20* | *0.02* |
| WCVI | 9523 | 0.22 | 0.24 | 0.81 | 0.76 | 0.87 | 0.03 | 0.21 | 0.00 |
| | | | *0.24* | *0.82* | *0.76* | *0.83* | *0.08* | *0.16* | *0.01* |

**Fig. 2. Heatmaps of model performance.** Class-based Accuracy, true negative rate (TNR) and Reliability for weighted (left column) and non-weighted (right column) models. The color shading within each row reflects the underlying numbers from high (red) to low (blue) and is included to enhance the differences between values.

The effect of weighting was notable in the error assessment where the Quantity errors of all non-weighted models (0.07 to 0.10) were about twice that of the corresponding weighted models (0.03 to 0.06) (Table 3). The majority of model error came from Exchange between classes. The reduction in Quantity error achieved by weighting tended to be offset by a corresponding increase in Exchange error. This explains why TSS and Accuracy were largely unchanged by weighting (Table 3).

The reduced Quantity error due to class weighting was also evident when the prevalence of the build testing partition was compared to model predictions (Fig. 3). The over-prediction of the Rock class by the non-weighted models, as well as the consistent under-prediction of the Mixed class, were clearly mitigated by class weighting. Changes in the prevalence of the Sand and Mud classes were more equivocal. More importantly, these changes in predicted prevalence were evident in small but significant shifts in the spatial distribution of the classes, with the weighted model producing a pattern that more accurately reflected known nearshore substrate in our two test locations (Fig. 4). The predictions from the weighted model produced less Rock and more Sand on known beaches in Pacific Rim. Changes in English Bay were less apparent, but a shift from Rock to Mixed is evident. In deeper waters of Pacific Rim, the weighted model predicted less Sand and more Mixed substrates, although bathymetric artefacts were enhanced.

**Fig. 3. Comparison of class prevalence.** Observed class prevalences in the build testing partition (orange) compared to predictions from the weighted (yellow) and unweighted (blue) random forest models across regions. Weighting tends to yield class prevalence closer to that observed in the training sample.

**Fig. 4. Predictions in regional assessment areas.** Predictions from the 100 m coastwide (top row), 20 m, no-weight (middle row), and 20 m weighted (bottom row) models for the Pacific Rim National Park (left column) and Greater Vancouver (right column) assessment areas. The series of images shows how increased resolution and weighting for prevalence help mitigate the bias towards hard substrate.

Variable importance (Fig. 5) differed notably across models despite similarities in the aggregate metrics (Table 4), suggesting different processes were at play. Our indicators of ocean dynamics (circulation and tidal) were the top two predictors for the coastwide model, followed by bathymetry. Slope, broad- and medium-BPI, and the standard deviation of slope provided almost equal contributions. In contrast, bathymetry was the dominant predictor for all regional models except HG, where bathymetry was second to fetch. Fetch was also important in the WCVI region, the other region we presumed would be influenced by exposure. Fetch, broad-BPI, and tidal flow rounded out the top four variables for the regions. Despite such rankings, the contributions of predictors can be very similar, particularly among those contributing least. For example in the NCC region, 4 of the least influential predictors have virtually equal model contribution scores (Fig. 5).

**Table 4: Aggregated build metrics for the Coast model and 100 m models built for the spatial extents of each of the 5 regions showing that at this resolution, there is no detectable difference between regions, and no difference between the regions and the coastwide model.**

| Region | N* | Imbalance | OOB | TSS | Accuracy | TNR | Quantity | Exchange | Shift |
|---|---|---|---|---|---|---|---|---|---|
| Coast | 66056 | 0.17 | 0.3 | 0.79 | 0.70 | 0.86 | 0.03 | 0.25 | 0.02 |
| HG | 9456 | 0.15 | 0.3 | 0.78 | 0.68 | 0.86 | 0.05 | 0.26 | 0.01 |
| NCC | 25873 | 0.20 | 0.3 | 0.78 | 0.70 | 0.84 | 0.04 | 0.24 | 0.02 |
| WCVI | 10205 | 0.21 | 0.3 | 0.80 | 0.73 | 0.85 | 0.04 | 0.22 | 0.02 |
| QCS | 4565 | 0.19 | 0.3 | 0.75 | 0.67 | 0.80 | 0.07 | 0.23 | 0.03 |
| SOG | 16025 | 0.13 | 0.3 | 0.80 | 0.71 | 0.89 | 0.02 | 0.27 | 0.00 |

* Regional sample sizes differ slightly from Table 1 because for this comparison the build data were separated using a regional shape file, whereas in Table 1 the points were selected using regional 20 m rasters.

**Fig. 5. Heatmap of variable importance across models.** Variable importance, defined as the proportion of each predictor's contribution to the model, is shown relative to the predictor with the highest contribution (p/max(p)). The color-shading within each row reflects the underlying numbers from high (red) to low (blue) and is included to make the differences in the values more apparent. NA indicates that Fetch was not used as a predictor for the Coast model.

## Model resolution

The most significant predictors for the 100 m coastwide model were related to ocean energy, at a native scale of 3 km, while the regional 20 m models were closely tied to bathymetry and potential wave energy. Yet comparing the 100 m and 20 m models shows little difference in either the aggregated or class-based metrics between resolutions (Table 4, Fig. 2), and there is no suggestion the 100 m model is skewed towards any particular region (Table 4). However, the differences in mapped predictions are dramatic, with the 100 m model showing an over-prediction of nearshore Rock in both focal areas, despite class weighting (Fig. 4). The mapped predictions from the 100 m model are also more homogeneous than the 20 m models, which reduces artefacts but also highlights the inability of the coarser model to represent local heterogeneity.

## Performance across depths

The 100 m model shows a clear trend of increasing TSS with deeper water compared to the 20 m models based on the testing partition, although results were variable across regions (Fig. 6a). Specifically, the trend is clear for HG and NCC, absent for SOG, and uneven for WCVI and QCS. This pattern is driven by the higher Accuracy of the 20 m models in the intertidal and 0-5 m depth zones (Fig. 7a) across all regions, except the SOG intertidal. The 100 m model has a consistently higher TNR, particularly in the 0-5 m depth zone (Fig. 6b). This can be attributed to the large, homogeneous Rock predictions of the 100 m model in shallower waters (Fig. 4). This allows the model to benefit from the higher (0.75) no-information baseline (compared to the 20 m model).

**Fig. 6. Differences in model fit by region, across depths and resolutions.** The (A) true skill statistic (TSS) and (C) true negative rate (TNR) between the 20 and 100 m models are shown by region, across depth ranges, using the build testing partition. Values below 0 indicate a higher

score by the 20 m model. This illustrates how regional model performance is generally better across all depths and regions, except for SOG, and a possible sampling biases in the 0-5 and 200+ depth ranges.

**Fig. 7. Comparison of error metrics by region, across depths and resolutions.** Error metrics for the 100m Coastwide (A) and 20 m regional models (B) are shown across depth zones, for each region using the build testing partition. Clear but noisy trends are apparent for increased accuracy with depth by the 100 m model and a decreased accuracy with depth by the 20 m models.

The corresponding error assessment for the two resolutions also shows Accuracy increasing with depth for the 100 m model across most regions (Fig. 7a) but generally decreasing with depth for the regional models (Fig. 7b). Across all models, there is a tendency towards increased Quantity error with depth, but most of the error is from an Exchange between classes.

The role of resolution in the correlation between model performance and depth is further supported by the IDE (see below), and is also apparent in the mapped predictions (Fig. 4, Fig. 8). The tendency of the 100 m model to predict more contiguous classes, and over predict Rock near shore is evident in all three of our test regions (Fig. 4e, f, Fig. 8b). However, the 100 m model also captures known features in deeper waters, in particular the canyons in Queen Charlotte Sound and the shelf edge not identified by the 20 m models (Fig. 8).

**Fig. 8. Mapped predictions for different resolutions.** Predictions for A) two 20 m regional models and B) the 100 m coastwide model are shown. Note the detail provided by the 20 m models near shore where the 100 m model predicts largely Rock. In contrast, the 100 m model identifies known features at depth (e.g., Moresby Canyon) not captured by the 20 m models. The regional models shown here were produced using unclipped predictor variables to allow model performance in deeper waters to be examined. See text for details.

## Independent data evaluation

The independent data were not consistently distributed across regions: the Dive data were distributed most broadly, while the ROV data were limited to HG and NCC (Table 5, Fig. S1). Not unexpectedly, model performance scores were generally lower and more variable than for the build testing data. The TSS for the 100 m coastwide model was similar (0.53 to 0.56) against all three independent data sets (Table 5). All regional models scored higher TSS scores (> 0.60) against the Dive and ROV data, while for the Cam data scores varied by region. For each independent data set, accuracies were as much as 50% higher for the regional compared to the coastwide model (Table 5). The TNR scores were highest for the ROV data followed by the Cam data, and notably lower for the Dive data (Table 5).

**Table 5: Performance of each random forest model against each independent data set.**

| IDS | Model | N | Imbalance | TSS | Accuracy | TNR | Quantity | Exchange | Shift |
|---|---|---|---|---|---|---|---|---|---|
| Dive | Coast | 3666 | 0.22 | 0.60 | 0.52 | 0.57 | 0.31 | 0.15 | 0.02 |
| | HG | 1479 | 0.23 | 0.66 | 0.59 | 0.76 | 0.07 | 0.30 | 0.04 |
| | NCC | 2217 | 0.30 | 0.66 | 0.65 | 0.57 | 0.19 | 0.14 | 0.03 |
| | WCVI | 166 | 0.36 | 0.69 | 0.69 | 0.58 | 0.16 | 0.11 | 0.04 |
| | QCS | 549 | 0.27 | 0.64 | 0.59 | 0.64 | 0.16 | 0.20 | 0.05 |
| | SOG | 551 | 0.14 | 0.64 | 0.48 | 0.77 | 0.22 | 0.24 | 0.06 |
| Cam | Coast | 2047 | 0.06 | 0.55 | 0.22 | 0.80 | 0.70 | 0.06 | 0.02 |
| | HG | 818 | 0.10 | 0.57 | 0.34 | 0.79 | 0.30 | 0.26 | 0.10 |
| | NCC | 580 | 0.06 | 0.60 | 0.39 | 0.76 | 0.34 | 0.16 | 0.11 |
| | WCVI | 139 | 0.20 | 0.55 | 0.22 | 0.87 | 0.58 | 0.17 | 0.03 |
| | QCS | 410 | 0.10 | 0.55 | 0.33 | 0.73 | 0.41 | 0.15 | 0.11 |
| | SOG | 196 | 0.21 | 0.55 | 0.24 | 0.86 | 0.55 | 0.14 | 0.07 |
| ROV | Coast | 6059 | 0.15 | 0.62 | 0.42 | 0.82 | 0.29 | 0.23 | 0.06 |
| | HG | 1762 | 0.14 | 0.66 | 0.39 | 0.87 | 0.48 | 0.11 | 0.02 |
| | NCC | 3909 | 0.18 | 0.63 | 0.48 | 0.79 | 0.13 | 0.36 | 0.03 |

Overall, the Dive and Cam data were predicted with the highest and lowest Accuracy respectively. Accuracy was inversely correlated with Imbalance, which (while variable across regions) was lowest for the Cam data and highest for the Dive data (Table 5). The lower TNR when predicting the Dive data is reflected in the Exchange error (Table 5), indicating that much of the misclassification is the swapping of two categories.

Examining the aggregated scores adjusted to their random baselines (Fig. 9) provided a more accurate representation of predictive power. The regional models generally outperformed the coastwide model (with adjusted TSS scores several times higher, and an Accuracy almost double) for the models (HG, NCC) with high sample sizes. For the other regions, the models predicted the Dive data better than the coastwide model, but accurate prediction of the Cam data was variable. The ROV data were predicted with the most consistent Accuracy and TNR scores, while the Dive data were consistently predicted with both the highest Accuracy and lowest TNR. These differences are at least in part due to the lower sample size, but may also reflect some spatial bias in the sampling.

**Fig 9. Integrated accuracy metrics of predictive power for each independent data set by region.** Accuracies are shown as the difference from the random baseline. Thus positive values indicate performance better than random and negative values indicate performance worse than random. In a multi-class error matrix, the random baseline differs for each statistic: for the true skill statistic (TSS) it is 0.5, for the true positive rate (TPR) it is 0.25, and for the true negative rate (TNR) it is 0.75. Where bars are not shown, the difference was 0, indicating random performance.

The influence of resolution on the correlation between depth and model performance is also apparent when the predictive power of the coastwide 100 m model is compared to the 20 m models for the regions with sufficient independent data (Fig. 10). The IDE of the coastwide model (Fig. 10a) shows a clear increase in predictive accuracy with depth for both the Dive and Cam

data, while the opposite pattern is evident in both the HG and NCC regions (Figs. 10a, b). The IDE of the ROV data are more equivocal, illustrating differences between independent data sets and emphasizing the need to understand the differences between the various data contexts.

**Fig. 10. Error assessment of three predictions, for each independent data set by depth zone.** A) Coastwide, B) Haida Gwaii, and C) North Central Coast models (regions not shown had insufficient sample size).

Such differences can be attributed to both data preparation or collection. In the first case, the occurrence of Dive data deeper than 50 m depth (as implied by the 100 m model, Fig. 10a) is an artefact of large pixels in the coastal zone over-generalizing depths. This effect is also evident in the ROV data, which are typically not collected in waters shallower than 20 m. This misallocation of the data is not due to positional inaccuracy of the observations because we screened the IDE data for coherence with the 20 m bathymetry. Rather, this is due to the resolution of the modelled bathymetry. Specifically, since a 100 m raster cell often covers a wide range of depths, particularly in steep topography, any associated observations would be assigned a single value, even if collected at different depths. Thus, what can appear to be a positional inaccuracy or bad data can actually be a function of analytic resolution.

# Discussion

Our results show that model reliability depends on depth, resolution, and substrate class, and potentially the uniqueness (in predictor space) of the location in question. This means reliability will vary across the coast, with different resolutions and substrate classes being relatively more or less reliable in different locations. Understanding these differences will improve the confidence that can be placed in these models, and their application to emergency response and predictions of habitat suitability. It will also help guide future data collection, and support model refinement.

## Depth and resolution

All our models fit the build data well, however the predictive power of the regional 20 m models was universally better than the coastwide model. Further, model performance was dependent on depth and resolution, confirming our initial belief that the 100 m model would perform better in deeper waters, and the 20 m models better in shallow waters. Our qualitative assessment of the mapped predictions agrees with the numerical analysis: the 100 m prediction of large contiguous areas of Rock nearshore, and the failure of the 20 m model to represent known features (e.g., canyons) in deeper waters (Figs. 4, 8) illustrate how coarser spatial resolutions are unable to represent finer scale heterogeneity in bottom structure, while the resolutions needed to capture that heterogeneity can miss larger features. A comprehensive mapping of the different scales of heterogeneity will therefore require features defined from multiple resolutions. The integration of these diverse features would be best captured using an object-based approach [e.g., 8, 14] where representation is not resolution-dependent.

Our results also support the view that substrate heterogeneity generally decreases with depth, and suggest that resolution-based differences in performance across depths (Figs. 6, 7) are influenced by the actual heterogeneity in different depth classes. For example, the higher accuracy of 20 m models in shallower water is because they can better capture nearshore heterogeneity. The more consistent fit of the 100 m model across depth classes in the SOG region can then be explained because the region is a marginal, relatively shallow sea dominated by mud in deeper waters [63] with a more homogenous coastline than the other, more exposed regions. This highlights the importance of considering process stationarity (see following section).

Nevertheless, when true heterogeneity does decrease with depth, it will be better represented by higher resolution near shore, and coarser resolutions in deeper waters. The 20 m models can

therefore be considered more appropriate for shallower areas, and the 100 m model better at resolving key features in deeper waters. More specifically, the correlation of predictive power with depth (Fig. 7) suggests that the 20 m models will be more reliable for nearshore studies to about 50 m depth, while the 100 m model would be more reliable in deeper areas.

To assess whether sampling density contributed to model performance, we examined model fit to data spatially, looking for patterns in accuracy (not shown). We found sample density to be highly correlated with depth (not surprising given the sampling context of much of the build data), making it impossible to disentangle the effect of density from depth with our available observations. While understanding the role of sample density would provide important information to the design of sampling programs, accuracy is likely to be maximized when sample density is the same or better than the analytic resolution. Ideally, choice of resolution would include an explicit rationale to ensure the resulting product is not mis-interpreted by drawing inferences at inappropriate resolutions.

Analytic resolution also influences process stationarity because processes are scale-dependent (Wiens 1989). This means coarser models will better represent more averaged conditions (which by definition have less variability and correspondingly higher stationary). Thus, our observed differences between the 20 m and 100 m models are also due, in part, to the different processes captured by the different resolutions.

## Process stationarity

Correctly representing driving processes is central to predictive power (Austin 2002). However, the reliability of such representations across a seascape depends in part on the assumption of stationarity, a typically tenuous assumption, particularly across larger extents [64]. In this analysis, both class-based results (Fig. 2) and variable importance (Fig. 5) showed strong evidence for non-stationary processes across regions, while non-stationarity across both regions

and depths is suggested by differences in aggregated metrics of model fit (Figs. 6, 7) and error assessment (Fig. 9).

There are obvious reasons for non-stationarity across regions. For example, the SOG is dominated by sedimentation processes (e.g., runoff), that in more exposed regions will have a stronger interaction with water energy. Elsewhere, the unique characterization of variable importance on the NCC likely reflects its geomorphic context, where the competing dominance of tidal energy in channels and wind-wave energy (fetch) in inlets creates within-region differences. Finally, predictions of sand in exposed coastal areas of the WCVI may be made more difficult because very local processes differ from an otherwise strong association between Rock and high fetch. Other (unrepresented) factors such as freshwater input and upland geology will also differ both within and across regions. Given that these processes play out on a crenulated coastline carved up by deep narrow fjords, it’s not surprising they depend on local context.

Such local processes cannot be teased out by classification models. Instead, they are generalized across the model domain according to the prevalence and spatial distribution of the observations. We argue that model reliability is higher in places where predictor values are closer to the center of their ranges, thereby avoiding boundary conditions. This means oceanographic uniqueness will likely be correlated with poor model quality.

## Bias and accuracy

Models of substrate, habitat and climate are regularly built on other models. Thus, any layers used in this way (e.g., bathymetries, current models, remotely sensed primary production) will have their own artefacts, uncertainties and limitations. Careful consideration of data sampling and processing steps such as averaging, aggregation, or interpolation methods is therefore warranted. This is most evident in this study by the assignment of over-generalized depths to the

IDE dive and ROV data (Fig. 10) and illustrates the need for analysts to consider how resolution may influence the conclusions that can be drawn.

In contrast to their consistent validation against the build test data, the predictive power of our models varied across the independent data sets, a clear indication of the different sampling contexts. This suggests that building models with a compilation of data from different sampling contexts can reduce (or average) sampling bias, further implying that a model built with data from a single sampling context may have lower predictive power. We suggest that using build data compiled from several sampling contexts will improve model performance because the diversity of sampling biases will force the classifications to be more general, much like the generalization of processes described above regarding stationarity.

Model reliability is also influenced by the number and nature of the classes used in the classification. For example, Rock, Sand, and Mud are all more definitive than a mixed class, which by definition includes a variety of heterogeneous classes (e.g., sand and cobble, gravelly sand, boulders on silt). With this diversity (i.e., a lack of independence), any method would be hard-pressed to reliably describe this class. So it's not unexpected mixed was less well predicted than the more independent classes (see Table S4). We suggest such a class is useful to the overall classification because providing a home for less definitive observations reduces the misclassification of the other classes, and provides insight into local heterogeneity. While an alternative is to have more classes, this can exacerbate the prevalence problem.

Spatially, we have shown that examining mapped predictions is also critical to understanding model accuracy. Our qualitative visual assessment identified both poor spatial accuracy in mapped predictions and spatial artefacts, neither of which are apparent in the performance metrics. In addition to non-stationarity and analytic resolution (described above), the accuracy of predictors must also be considered. For example, the ocean current model we used (for all models except the 20 m SOG) had a native resolution of 3 km. While the associated predictors

(tidal and ocean current energy) were the most important predictors in the 100 m model, the downscaling to 20 m may have served to obscure rather than enhance nearshore variability, despite still helping explain the broader pattern in the observations. Similarly, the artefacts evident in both the 20 and 100 m bathymetric models, themselves the result of sampling bias [e.g., 65] serve as a caution to fitting models too closely to modelled output. These insights suggest we have reached the limits of what can be achieved with our existing predictor data, and that higher resolution predictors that more closely match the analytic resolution are likely to improve model accuracy.

Recognizing artefacts is critical. We suggest all predictor layers be examined carefully before modelling. Our experience shows that artefacts (e.g., for depth) are more clearly visible when examining derivatives. If artefacts are observed, they can often be mitigated by smoothing (Fig. S3). Another example relates to the importance of land-side elevations, which are beneficial when generating derivatives in coastal systems. In their absence, the edge effect caused by absent elevations above the intertidal zone can lead to underestimates of intertidal slope and rugosity [65], and errors in the derivation of other subsequent predictors. Using the terrestrial data in the creation of our predictors improved the representation of steep-sided rocky shorelines (e.g., Fig. S4), a shortcoming noted by Haggarty and Yamanaka (2018).

Finally, examining the role and implications of sample prevalence is critical given the challenges imbalanced data pose to random forest models [51]. Despite the imbalance in our build data, the mapped predictions were more balanced (Fig. S2) and more consistent with the known characteristics of these regions [63] and our collective experience with the study area. This is reassuring. However, IDE suggests that differences in prevalence between the build and independent test data could influence estimates of predictive power. Specifically, our models predicted the Dive data (which, like the build data, were biased towards Rock substrate) with greater accuracy than the more balanced Cam data. Differences in prevalence between build

and test data could therefore influence estimates of predictive power. While we found no guidance on whether model accuracy improves with increasing agreement between sample and true prevalence. This highlights the importance of methods to address this imbalance, and leads to the question of how to trade off accuracy based on a balanced training sample against how accurately the real-world prevalence of the classes is captured.

## Measuring performance

Our study shows the value of using both accuracy and error assessment metrics, comparing performance across spatial subsets (e.g., regions and depths), and class-based metrics. The error assessment provided insight into tests of predictive power, much like the ground-truthing of models in a remote sensing context. Class-based metrics showed the effect of class weighting on model performance (Fig. 2), and contributed to our assessment of spatial non-stationarity (Table 5, Fig. 9) by identifying differences between regions and the effect of depth on the performance of different model resolutions. By using well known focal areas, our qualitative spatial assessment uncovered important differences between numerical and mapped performance not otherwise apparent. Other, more detailed, spatial assessments are possible, but as in our case they would be complicated by spatial sampling bias (e.g., the hard bias of the training data, and the shallow bias of the independent dive data), sample density, and questions of spatial-autocorrelation. Such analyses would therefore be most effective with purpose-collected data.

## Next steps

Our illustration of how depth and resolution influence predictive performance challenges the feasibility of producing a gridded coastwide substrate map at a single resolution, pointing to the need for an object-based framework to integrate substrate class polygons developed at multiple resolutions. Such efforts could extend existing object-based efforts in the region [14], and take

advantage of the work done testing the performance of predictors across resolutions done in the context of random forest BS classification [21].

Independent data collected with dedicated surveys would not only support tests of spatial error, but could be used to test the suggestion that more typical parts of the coast will be better predicted than more unique areas. Such spatially unbiased data could also be used to assess sub-regional differences in process. Improving predictor resolution would also help improve classification in shallower waters. For example, the interpolated fetch used in some of the 20 m regional models could be replaced with a grid of fetch values, while all models would likely benefit from a coastwide ocean circulation model with a sub-kilometer resolution.

Methodologically, more work is needed on understanding the role of sample imbalance because while weighting did provide some correction for prevalence, our models continue to over-predict hard substrate. Disentangling the effect of sample size from its spatial distribution is also an understudied challenge. Questions include 1) Is higher accuracy achieved with a balanced training sample or one that more closely corresponds to the real-world prevalence? 2) Is model accuracy higher in areas with higher sampling density? And 3) Is accuracy maximized by matching the analytic and sampling resolution? Answers to these questions would improve sampling design and support the development of an object-based approach to integrate features from different resolutions.

Investigating how the performance of a boundary class (like Mixed in this analysis) could help guide model refinement may also be worth investigating. To understand the interaction between sampling density and depth, estimates of spatial variability in the training data [e.g., 66], or spatial uncertainty in model predictions [e.g., 2] could provide some insights. Such methods may, however, be more relevant to the ecological models produced from the substrate layer presented here.

Despite the significant challenges facing the classification of BS data collected during bathymetric surveys, meter-scale bathymetry for an increasingly large portion of the coast is becoming available, particularly in deeper waters. These data could be used directly in our analysis at local scales to produce higher resolution outputs and support cross-scale analyses. More detailed, reliable, local MB classifications calibrated to BS data [e.g., 30] would be invaluable as independent data to test the substrate predictions developed here

# Conclusions

We have produced a set of comprehensive, coastwide maps of marine substrate at resolutions appropriate to nearshore and coastwide analyses. Our spatial assessment shows that our 20 m regional models are suitable for shallower (< 50 m depth), coastal regions, while our 100 m model is more suitable for deeper, more homogenous areas of the shelf. Although higher resolution (e.g., meter-scale) models are feasible, they will require higher resolution predictors, and will likely have to be limited to regional or sub-regional areas to manage the challenge of stationarity.

We expect model reliability will be highest in more typical, well-sampled areas of the coast, where predictor values were closer to the coastwide or regional average. Predictions in more unique, under-represented areas will be less reliable. Understanding the relationship between resolution and representable features will help users assess the reliability of the mapped predictions.

Our tests of predictive power suggest that building models with data compiled from diverse sampling contexts may improve predictive power by integrating the sampling biases into the models. They also emphasize the importance of distinguishing predictive power from model validation.

Our analysis is one of the few to predict substrate classes from a diverse set of observations over large spatial extents at ecologically relevant scales. Our predictive models are also the first to be evaluated using both accuracy and error metrics to understand model performance, showing the benefits of comprehensive model assessment. Our maps will contribute to marine spatial planning initiatives in Pacific Canada, and our methods may be useful in other jurisdictions where substrate maps are required.

# Acknowledgements

This work was funded by the World Class Oil Spill Response initiative overseen by the Department of Fisheries and Oceans. Matt Grinnell created the fetch points in Hecate Strait and Queen Charlotte Sound. Input and support by Jessica Finney and collegial reviews of an earlier draft by Spencer Cooper and Beatrice Proudfoot greatly improved the coherence and readability of this paper.

# References

1. Gregr EJ, Baumgartner MF, Laidre KL, Palacios DM. Marine mammal habitat models come of age: the emergence of ecological and management relevance. Endangered Species Research. 2013;22:205-12.
2. Nephin J, Gregr EJ, St. Germain C, Fields C, Finney JL. Development of a Species Distribution Modelling Framework and its Application to Twelve Species on Canada's Pacific Coast. In: Canada FaO, editor. Ottawa ON: Fisheries and Oceans Canada, Canadian Science Advisory Secretariat; 2020. p. xii + 107 p.
3. Harris PT, Baker EK. GeoHab atlas of seafloor geomorphic features and benthic habitats–synthesis and lessons learned. Seafloor Geomorphology as Benthic Habitat: Elsevier; 2020. p. 969-90.
4. Brown CJ, Smith SJ, Lawton P, Anderson JT. Benthic habitat mapping: A review of progress towards improved understanding of the spatial ecology of the seafloor using acoustic techniques. Estuar Coast Shelf Sci. 2011;92(3):502-20.
5. Kostylev VE, Todd BJ, Fader GB, Courtney R, Cameron GD, Pickrill RA. Benthic habitat mapping on the Scotian Shelf based on multibeam bathymetry, surficial geology and sea floor photographs. Mar Ecol Prog Ser. 2001;219:121-37.
6. CBD. Strategic Plan for Biodiversity 2011-2020, including Aichi Biodiversity Targets. In: Diversity CoB, editor. 2020.
7. UK. Global Ocean Alliance: 30 countries are now calling for greater ocean protection: Department for Environment, Food & Rural Affairs; 2020 [updated 3 October 2020; cited 2020 Nov 30 2020]. Available from: https://www.gov.uk/government/news/global-ocean-alliance-30-countries-are-now-calling-for-greater-ocean-protection.
8. Lawrence E, Hayes KR, Lucieer VL, Nichol SL, Dambacher JM, Hill NA, et al. Mapping habitats and developing baselines in offshore marine reserves with little prior knowledge: a critical evaluation of a new approach. PloS one. 2015;10(10):e0141051.
9. Misiuk B, Diesing M, Aitken A, Brown CJ, Edinger EN, Bell T. A spatially explicit comparison of quantitative and categorical modelling approaches for mapping seabed sediments using Random Forest. Geosciences. 2019;9(6):254.
10. Stephens D, Diesing M. Towards quantitative spatial models of seabed sediment composition. PloS one. 2015;10(11):e0142502.
11. Brown CJ, Blondel P. Developments in the application of multibeam sonar backscatter for seafloor habitat mapping. Applied Acoustics. 2009;70(10):1242-7.
12. Stephens D, Diesing M. A comparison of supervised classification methods for the prediction of substrate type using multibeam acoustic and legacy grain-size data. PloS one. 2014;9(4):e93950.
13. Lurton X, Lamarche G, Brown C, Lucieer V, RIce G, Schimel A, et al. Backscatter measurements by seafloor-mapping sonars: guidelines and recommendations. A collective report by members of the GeoHab Backscatter Working Group. 2015;(May):1-200.
14. Gregr EJ, Lessard J, Harper J. A spatial framework for representing nearshore ecosystems. Prog Oceanogr. 2013;115:189-201.

15. Haggarty DR, Yamanaka L. Evaluating Rockfish Conservation Areas in southern British Columbia, Canada using a Random Forest model of rocky reef habitat. Estuar Coast Shelf Sci. 2018;208:191-204.

16. Lucieer V, Hill NA, Barrett NS, Nichol S. Do marine substrates 'look'and 'sound'the same? Supervised classification of multibeam acoustic data using autonomous underwater vehicle images. Estuar Coast Shelf Sci. 2013;117:94-106.

17. Lamarche G, Lurton X. Recommendations for improved and coherent acquisition and processing of backscatter data from seafloor-mapping sonars. Marine Geophysical Research. 2018;39(1-2):5-22.

18. Cabral RB, Gaines SD, Lim MT, Atrigenio MP, Mamauag SS, Pedemonte GC, et al. Siting marine protected areas based on habitat quality and extent provides the greatest benefit to spatially structured metapopulations. Ecosphere. 2016;7(11):e01533.

19. Elith J, Leathwick JR. Species distribution models: ecological explanation and prediction across space and time. Annual review of ecology, evolution, and systematics. 2009;40:677-97.

20. Diesing M, Stephens D. A multi-model ensemble approach to seabed mapping. J Sea Res. 2015;100:62-9.

21. Misiuk B, Lecours V, Bell T. A multiscale approach to mapping seabed sediments. PLoS One. 2018;13(2):e0193647.

22. Porskamp P, Rattray A, Young M, Ierodiaconou D. Multiscale and hierarchical classification for benthic habitat mapping. Geosciences. 2018;8(4):119.

23. Pelletier D, Selmaoui-Folcher N, Bockel T, Schohn T. A regionally scalable habitat typology for assessing benthic habitats and fish communities: Application to New Caledonia reefs and lagoons. Ecology and Evolution. 2020.

24. Gregr EJ, Palacios DM, Thompson A, Chan KM. Why less complexity produces better forecasts: An independent data evaluation of kelp habitat models. Ecography. 2018. doi: 10.1111/ecog.03470.

25. Haggarty DR, Shurin JB, Yamanaka KL. Assessing population recovery inside British Columbia's Rockfish Conservation Areas with a remotely operated vehicle. Fisheries Research. 2016;183:165-79.

26. Turner JA, Babcock RC, Hovey R, Kendrick GA. Can single classifiers be as useful as model ensembles to produce benthic seabed substratum maps? Estuar Coast Shelf Sci. 2018;204:149-63.

27. Vasquez M, Chacón DM, Tempera F, O'Keeffe E, Galparsoro I, Alonso JS, et al. Broad-scale mapping of seafloor habitats in the north-east Atlantic using existing environmental data. J Sea Res. 2015;100:120-32.

28. Costanza R, d'Arge R, De Groot R, Farber S, Grasso M, Hannon B, et al. The value of the world's ecosystem services and natural capital. Ecol Econ. 1998;25(1):3-15.

29. Spalding MD, Fox HE, Allen GR, Davidson N, Ferdaña ZA, Max Finlayson, et al. Marine Ecoregions of the World: A Bioregionalization of Coastal and Shelf Areas. Bioscience. 2007;57(7):573-83.

30. Herkül K, Peterson A, Paekivi S. Applying multibeam sonar and mathematical modeling for mapping seabed substrate and biota of offshore shallows. Estuar Coast Shelf Sci. 2017;192:57-71.

31. Colotelo AH, Goldman AE, Wagner KA, Brown RS, Deng ZD, Richmond MC. A comparison of metrics to evaluate the effects of hydro-facility passage stressors on fish. Environ Rev. 2017;25(1):1-11.

32. Davies SC, Bureau D, Lessard J, Taylor S, Gillespie GE. Benthic habitat mapping surveys of eastern Haida Gwaii and the North Coast of British Columbia, 2013-2015. In: Canada FaO, editor. 2018. p. vi + 24 p.

33. Guisan A, Zimmermann NE. Predictive habitat distribution models in ecology. Ecol Model. 2000;135:147-86.

34. Foody GM. Explaining the unsuitability of the kappa coefficient in the assessment and comparison of the accuracy of thematic maps obtained by image classification. Remote Sens Environ. 2020;239:111630.

35. Pontius RGJ, Millones M. Death to Kappa: birth of quantity disagreement and allocation disagreement for accuracy assessment. Int J Remote Sens. 2011;32(15):4407-29.

36. Fawcett TW, Higginson AD. Heavy use of equations impedes communication among biologists. Proceedings of the National Academy of Science. 2012;109(29):11735-9.

37. Zhang C, Chen Y, Xu B, Xue Y, Ren Y. Temporal transferability of marine distribution models in a multispecies context. Ecol Indicators. 2020;117:106649.

38. Guisan A, Thuiller W, Zimmermann NE. Habitat suitability and distribution models: with applications in R: Cambridge University Press; 2017.

39. Araújo MB, Anderson RP, Barbosa AM, Beale CM, Dormann CF, Early R, et al. Standards for distribution models in biodiversity assessments. Science advances. 2019;5(1):eaat4858.

40. Roberts DR, Bahn V, Ciuti S, Boyce MS, Elith J, Guillera-Arroita G, et al. Cross-validation strategies for data with temporal, spatial, hierarchical, or phylogenetic structure. Ecography. 2017;40(8):913-29.

41. Yates KL, Bouchet PJ, Caley MJ, Mengersen K, Randin CF, Parnell S, et al. Outstanding challenges in the transferability of ecological models. Trends Ecol Evol. 2018;33(10):790-802.

42. Comber A, Brunsdon C, Charlton M, Harris P. Geographically weighted correspondence matrices for local error reporting and change analyses: mapping the spatial distribution of errors and change. Remote Sensing Letters. 2017;8(3):234-43.

43. Foody GM. Local characterization of thematic classification accuracy through spatially constrained confusion matrices. Int J Remote Sens. 2005;26(6):1217-28.

44. Breiman L. Random forests. Machine learning. 2001;45(1):5-32.

45. Cutler DR, Edwards Jr TC, Beard KH, Cutler A, Hess KT, Gibson J, et al. Random forests for classification in ecology. Ecology. 2007;88(11):2783-92.

46. Prasad AM, Iverson LR, Liaw A. Newer classification and regression tree techniques: bagging and random forests for ecological prediction. Ecosystems. 2006;9(2):181-99.

47. Hastie T, Tibshirani R, Friedman J. Random forests. The elements of statistical learning: Springer; 2009. p. 587-604.

48. R Core Team. R: A language and environment for statistical computing. 2018. https://www.R-project.org/.

49. ESRI. ArcGIS. 2019. 10.4.

50. Wright MN, Ziegler A. ranger: A fast implementation of random forests for high dimensional data in C++ and R. Journal of Statistical Software. 2017;77(1):1-17. doi: 10.18637/jss.v077.i01.

51. Johnson JM, Khoshgoftaar TM. Survey on deep learning with class imbalance. Journal of Big Data. 2019;6(1):27.

52. Allouche O, Tsoar A, Kadmon R. Assessing the accuracy of species distribution models: Prevalence, kappa and the true skill statistic (TSS). J Appl Ecol. 2006;43:1223-32. doi: 10.1111/j.1365-2664.2006.01214.x. PubMed PMID: 6607.

53. Manel S, Williams HC, Ormerod SJ. Evaluating presence–absence models in ecology: the need to account for prevalence. J Appl Ecol. 2001;38(5):921-31.

54. Conway KW, Johannessen D. Appendix A: Geology. In Ecosystem overview: Pacific North Coast Integrated Management Area (PNCIMA). Edited by Lucas, B.G., Verrin, S., and Brown, R. Can. Tech. Rep. Fish. Aquat. Sci. 2667: vi + 54 p. 2007.

55. Congalton RG. A review of assessing the accuracy of classifications of remotely sensed data. Remote Sens Environ. 1991;37(1):35-46.

56. Pontius RGJ, Santacruz A. Quantity, exchange, and shift components of difference in a square contingency table. Int J Remote Sens. 2014;35(21):7543-54.

57. Jolliffe IT, Stephenson DB. Forecast verification: a practitioner's guide in atmospheric science: John Wiley & Sons; 2012.

58. Dunn DC, Halpin PN. Rugosity-based regional modeling of hard-bottom habitat. Mar Ecol Prog Ser. 2009;377:1-11.

59. Gregr EJ, Gryba R, Li M, Alidina H, Kostylev V, Hannah CG. A benthic habitat template for Pacific Canada’s continental shelf. 2016. p. vii + 37.

60. Masson D, Fine I. Modeling seasonal to interannual ocean variability of coastal British Columbia. Journal of Geophysical Research: Oceans. 2012;117(C10):C10019:1-14. doi: 10.1029/2012jc008151.

61. Soontiens N, Allen SE, Latornell D, Le Souëf K, Machuca I, Paquin J-P, et al. Storm surges in the Strait of Georgia simulated with a regional model. Atmosphere-Ocean. 2016;54(1):1-21.

62. Lessard J, Campbell A. Describing northern abalone, haliotis kamtschatkana, habitat: focusing rebuilding efforts in British Columbia, Canada. J Shellfish Res. 2007;26(3):677-86.

63. Thomson RE. Oceanography of the British Columbia coast. 1981.

64. Scales KL, Hazen EL, Jacox MG, Edwards CA, Boustany AM, Oliver MJ, et al. Scale of inference: on the sensitivity of habitat models for wide-ranging marine predators to the resolution of environmental data. Ecography. 2017;40(1):210-20.

65. Davies SC, Gregr EJ, Bureau D, Wills P. Coastal digital elevation models integrating ocean bathymetry and land topography for marine ecological analyses in Pacific Canadian waters. In: Canada FaO, editor. 2019. p. vi + 38 p.

66. Mitchell PJ, Downie A-L, Diesing M. How good is my map? A tool for semi-automated thematic mapping and spatially explicit confidence assessment. Environ Model Software. 2018;108:111-22.

# Supplemental materials

## S1 – Performance metrics

## S2 – Supplemental tables and figures

## S2. Analytical methods

Figure 1

Click here to access/download;Figure;Fig1.tif

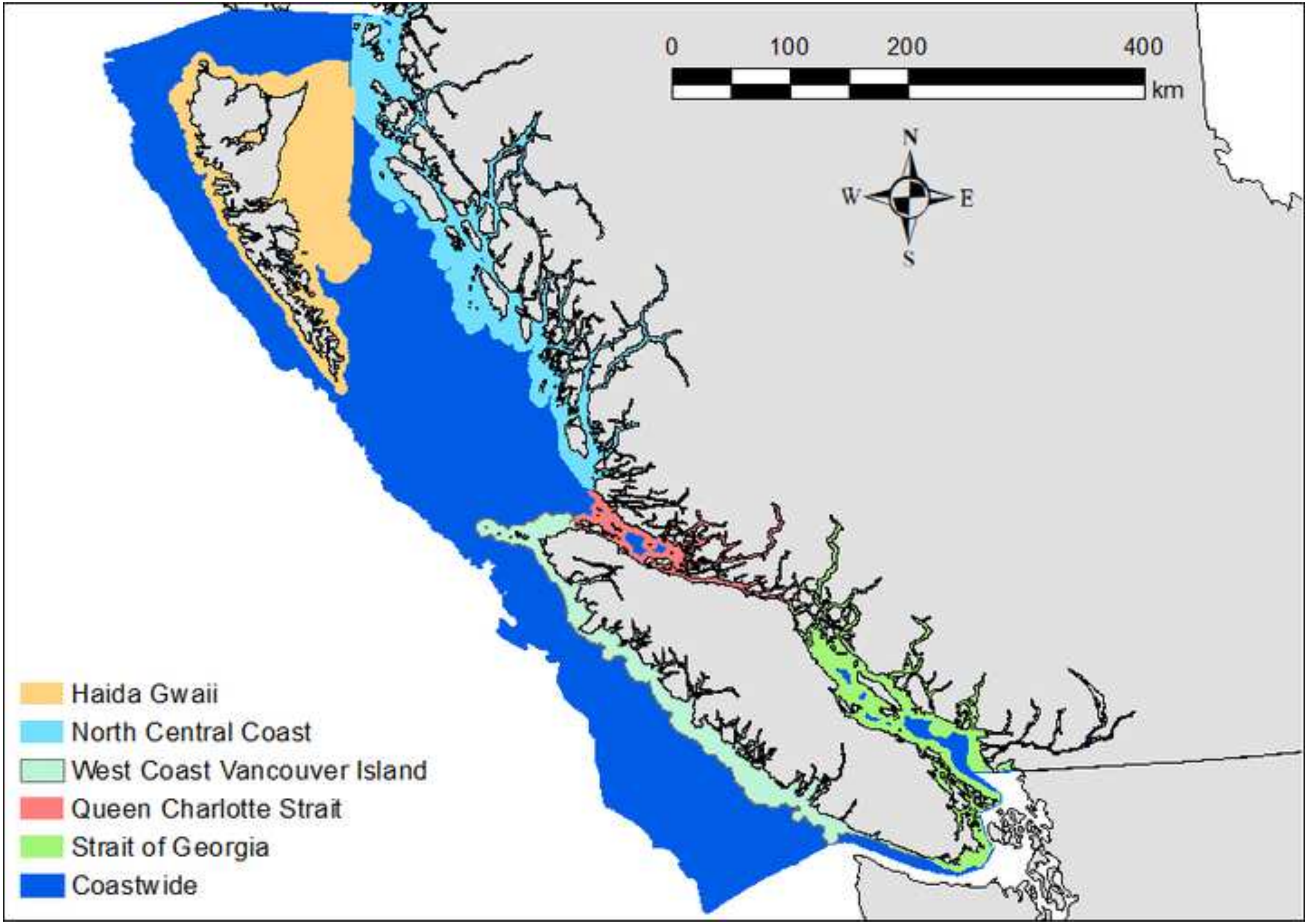

Figure 2

Click here to access/download;Figure;Fig2.tif

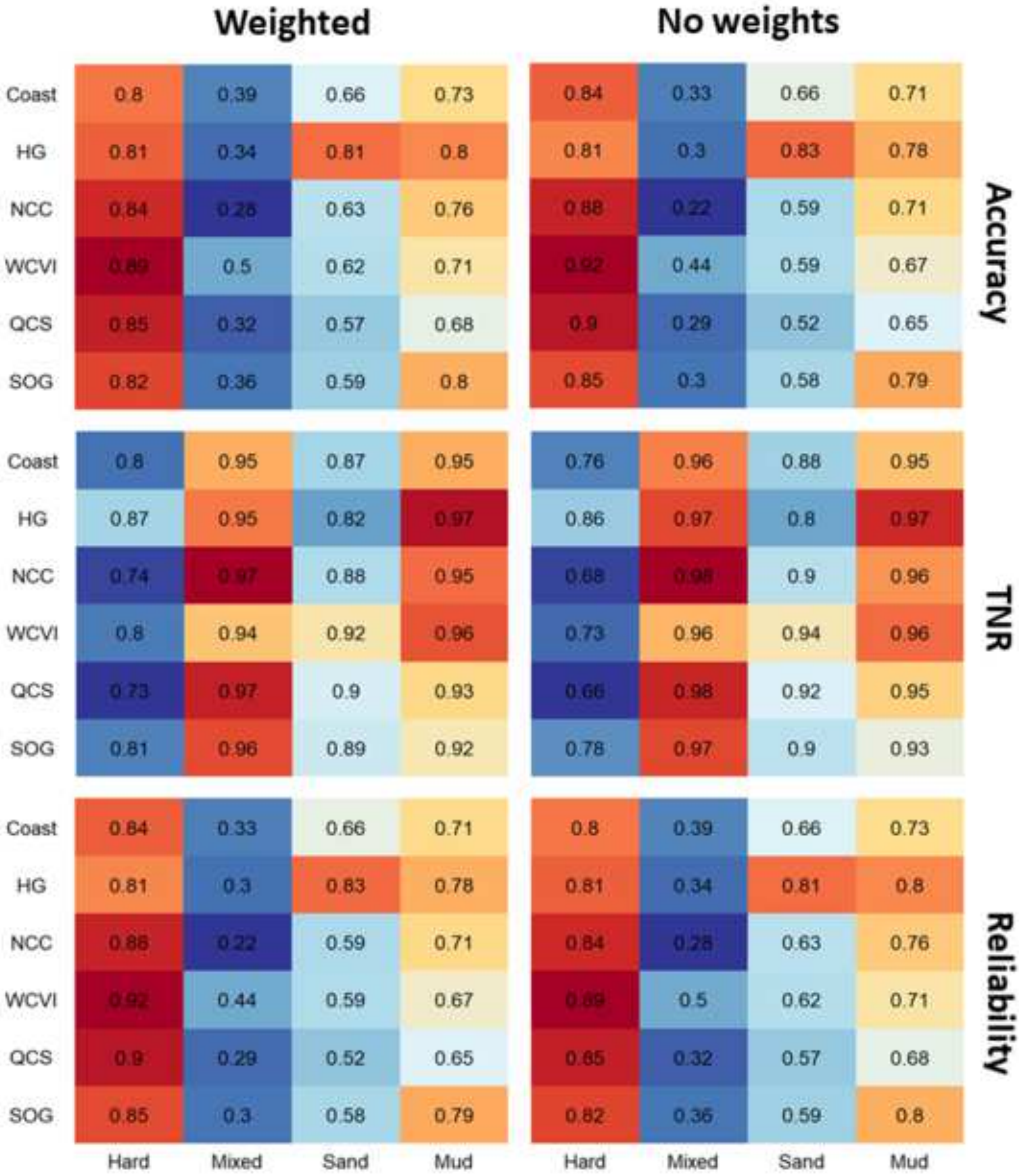

Figure 3

Click here to access/download;Figure;Fig3.tif

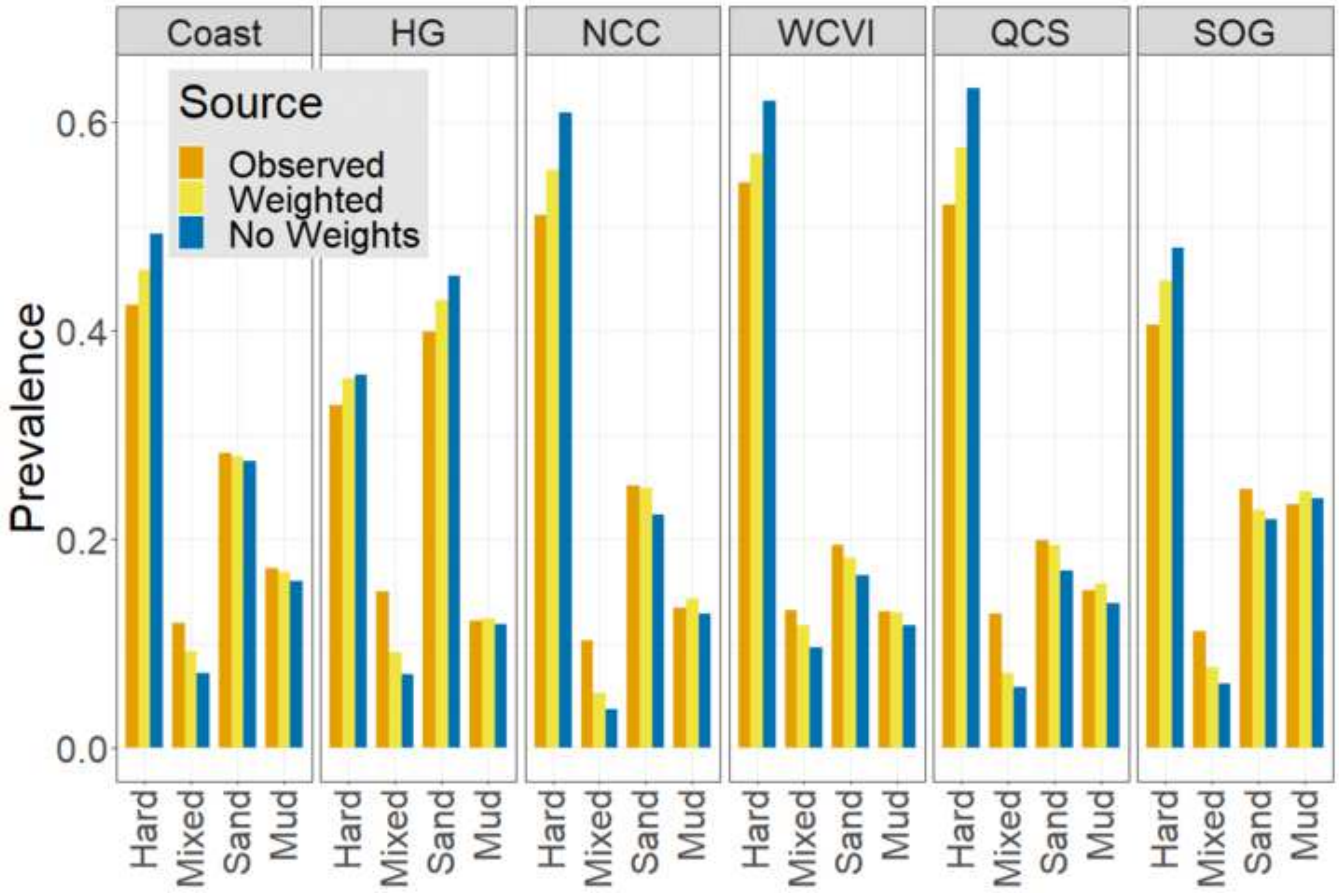

Figure 4 Click here to access/download;Figure;Fig4.tif

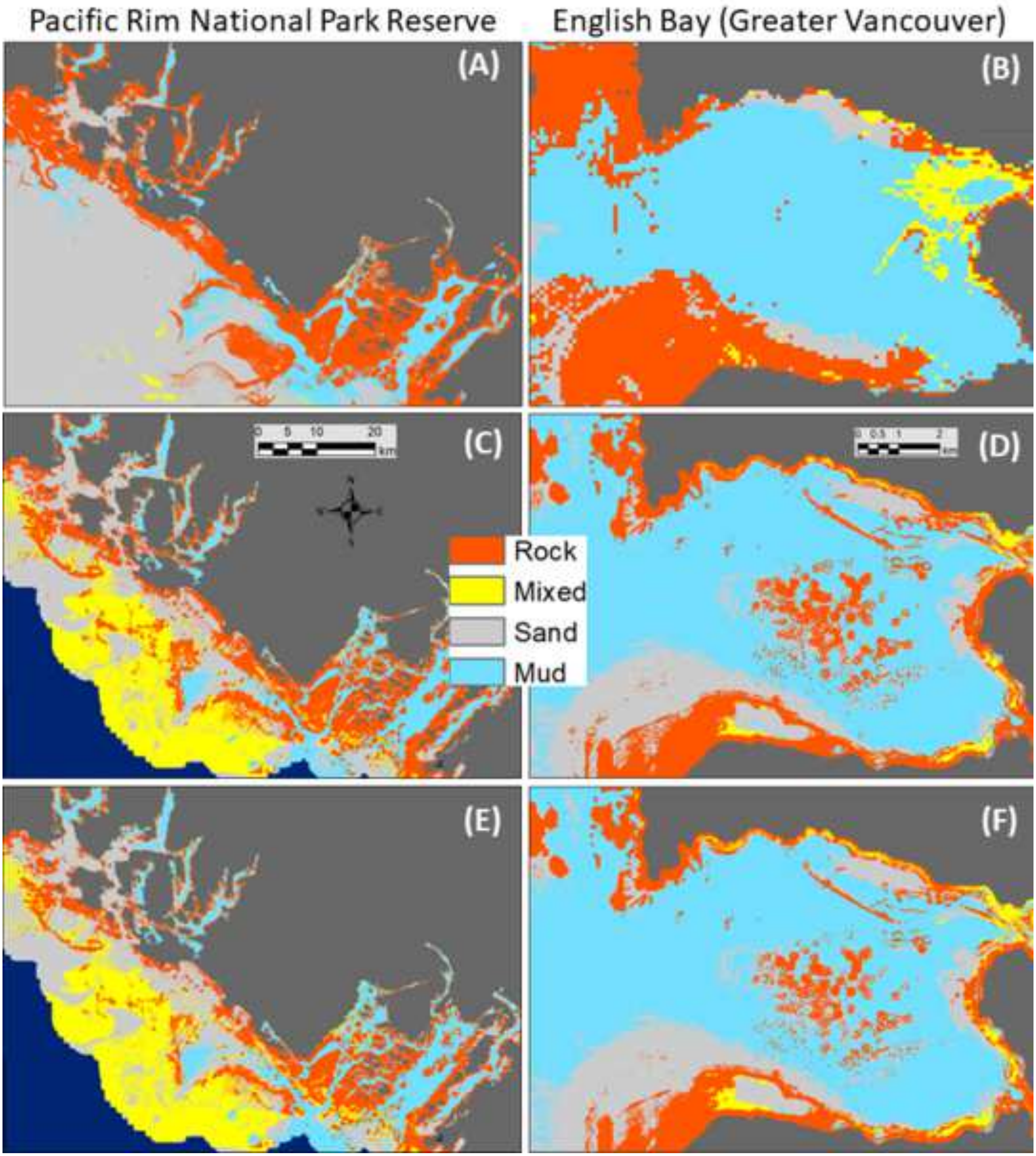

Figure 5

Click here to access/download;Figure;Fig5.tif

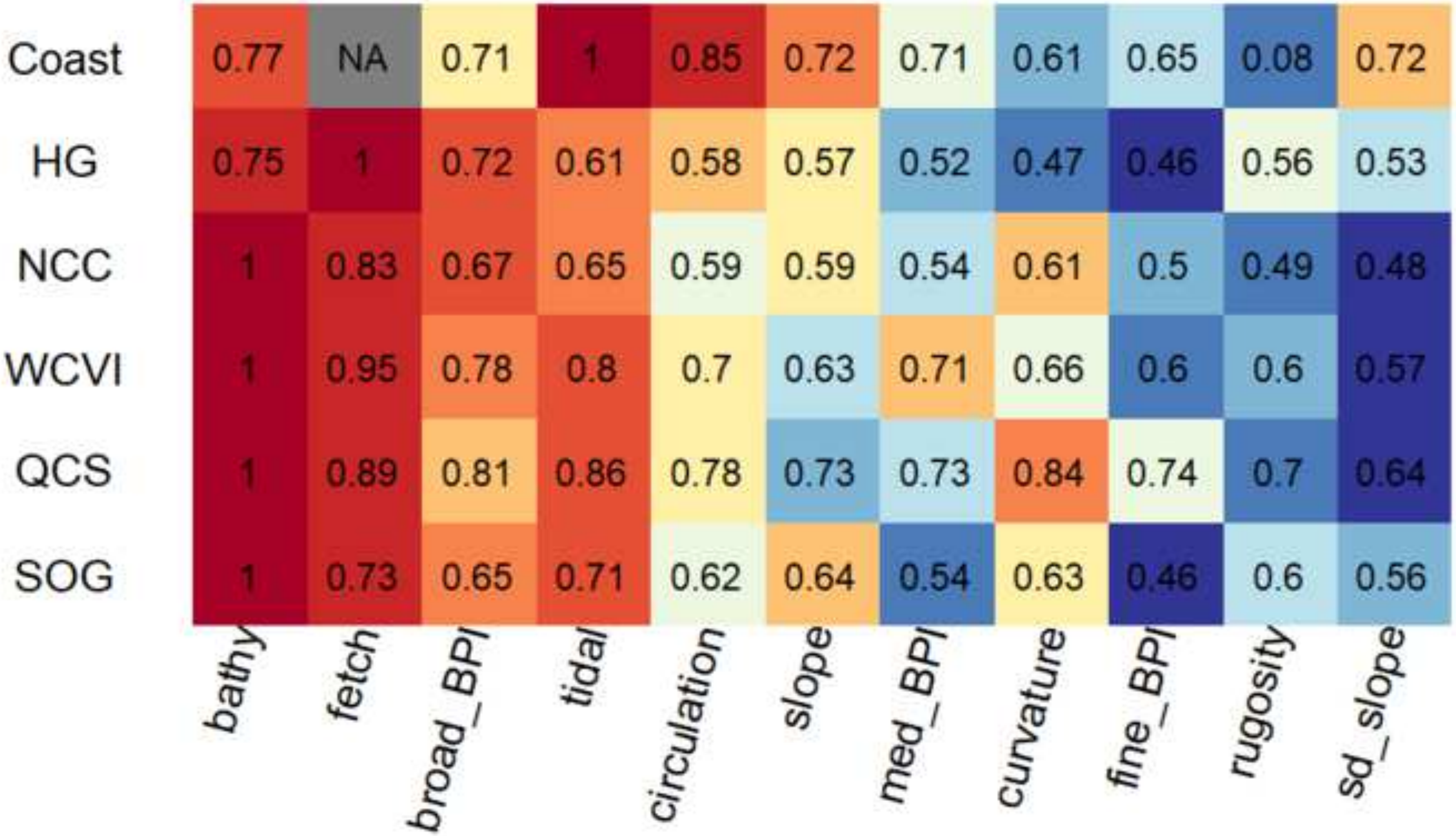

Figure 6

Click here to access/download;Figure;Fig6.tif

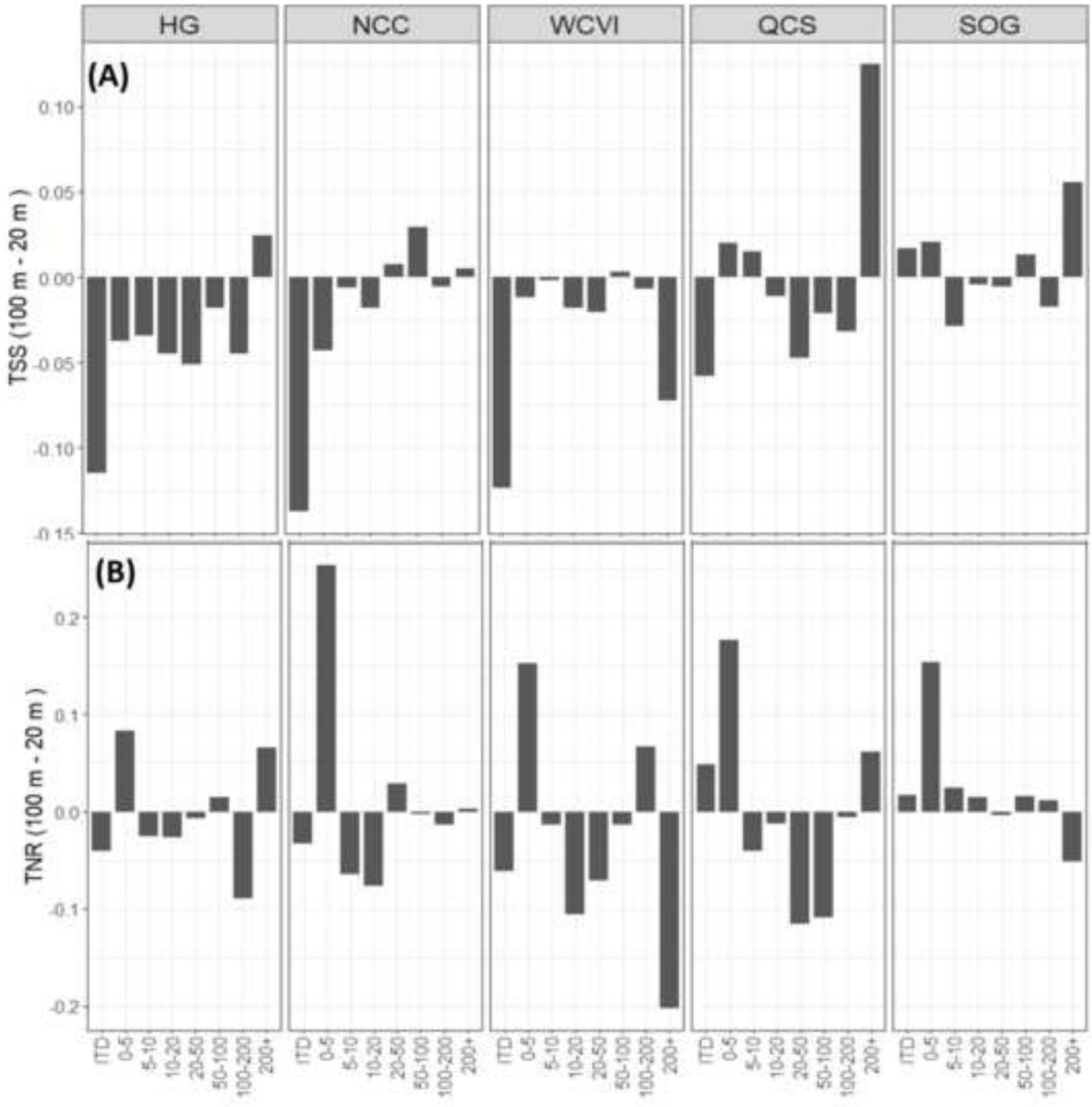

Figure 7 Click here to access/download;Figure;Fig7.tif

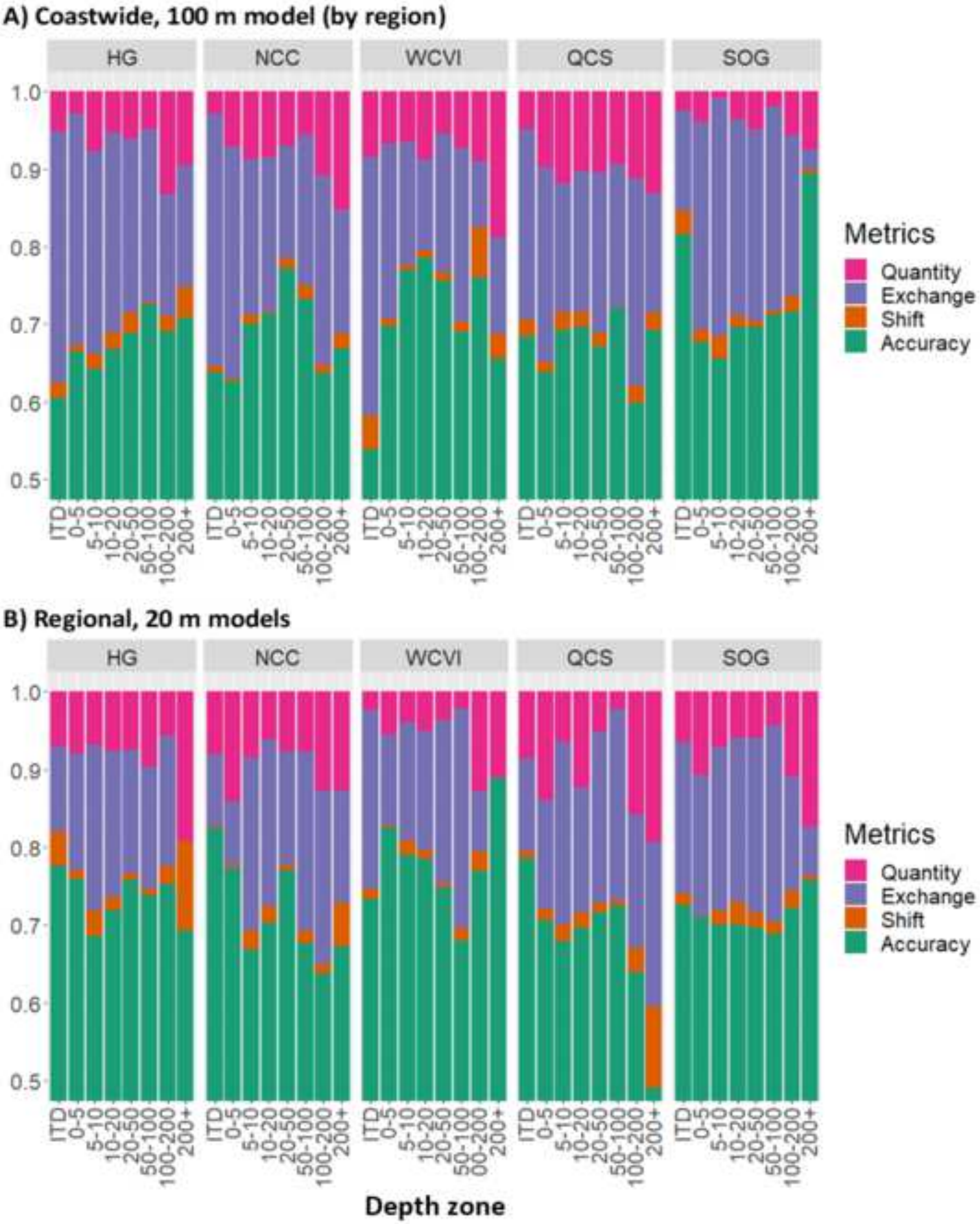

Figure 8

Click here to access/download;Figure;Fig8.tif

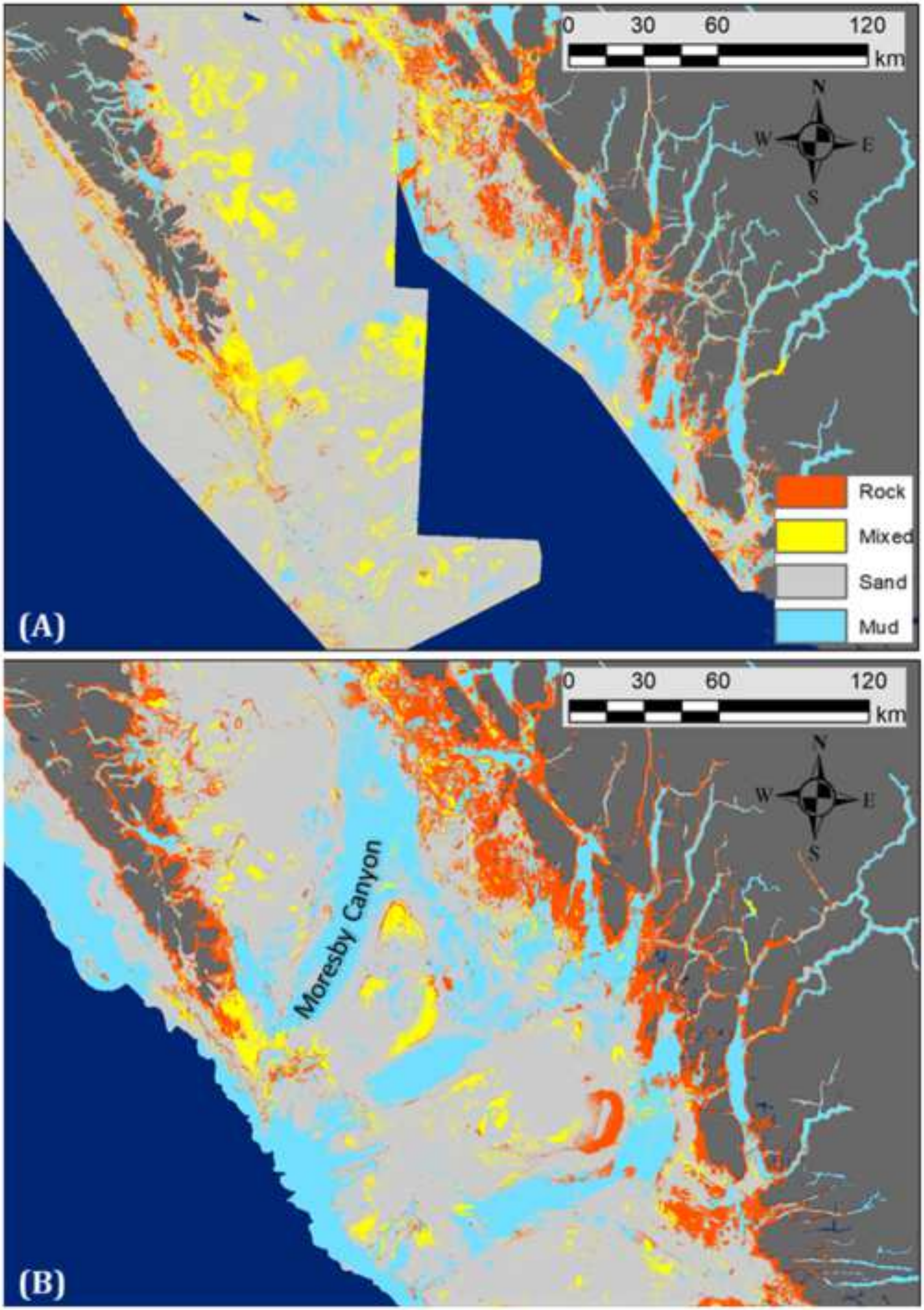

Figure 9

Click here to access/download;Figure;Fig9.tif

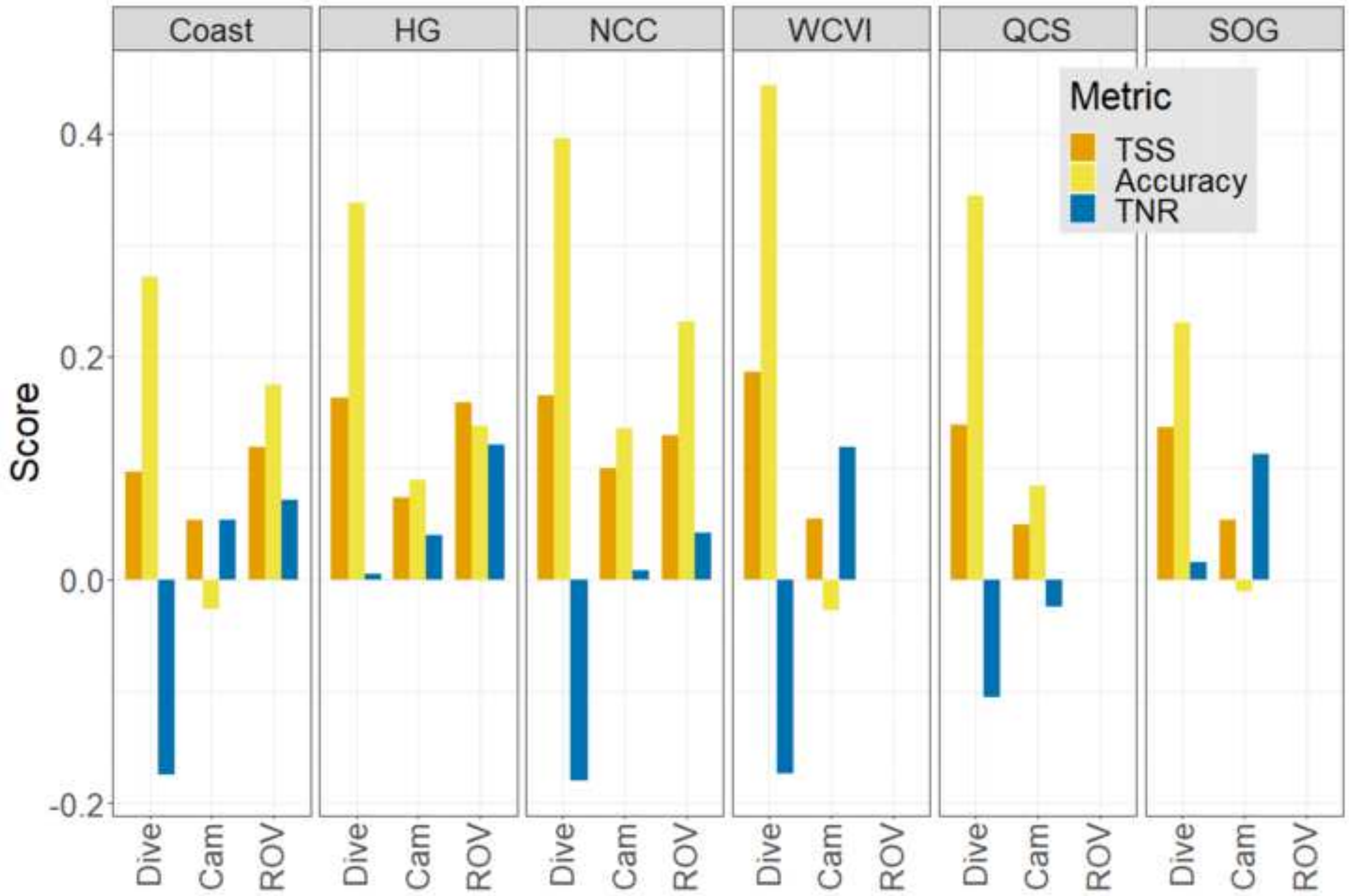

Figure 10

Click here to access/download;Figure;Fig10.tif

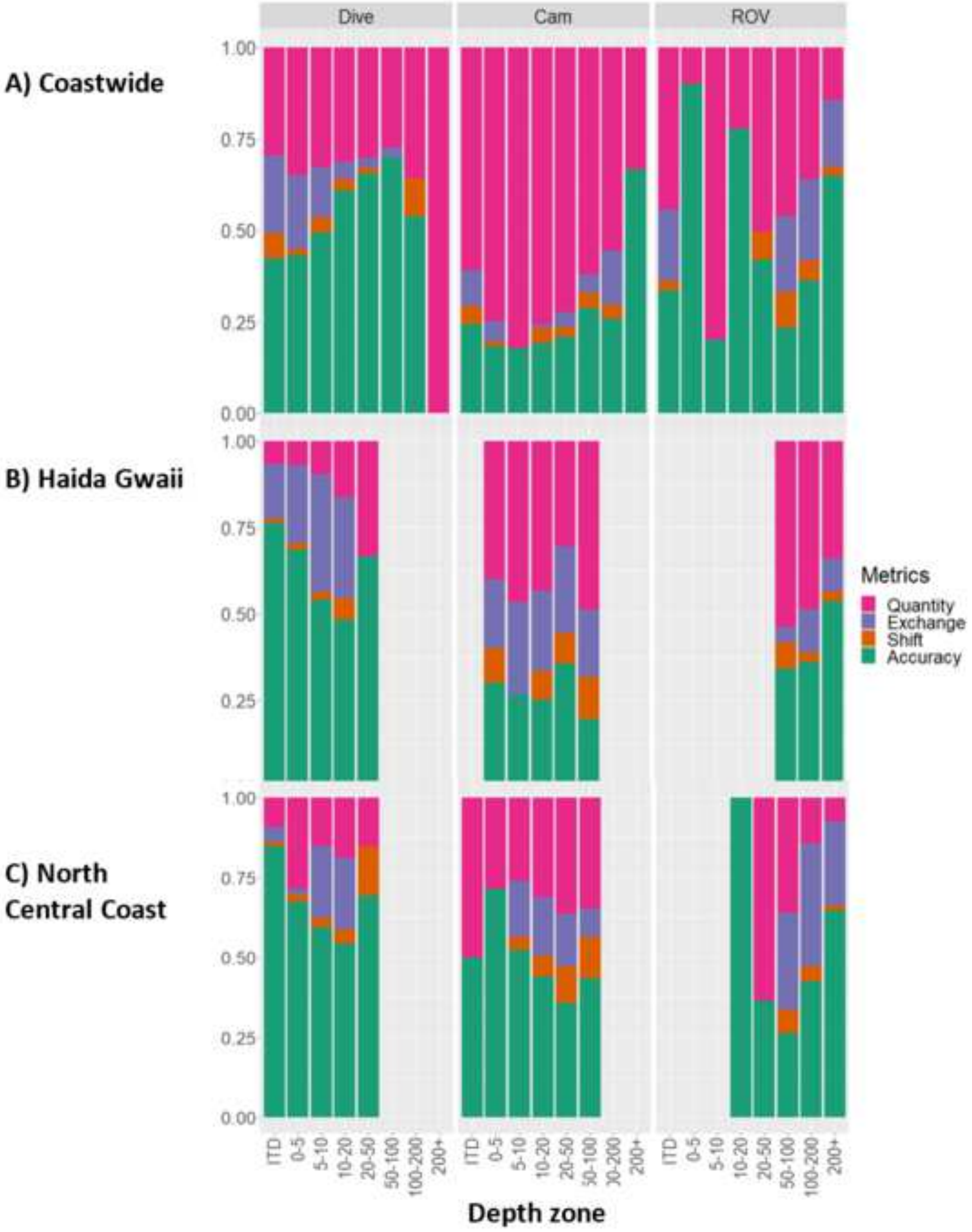

Click here to access/download
**Supporting Information**
substrate paper Supp Info version 3.2_PLOS.docx
