# Supplementary material for: Comprehensive marine substrate classification applied to Canada’s Pacific shelf
Source: PLoS One. 2021 Oct 29;16(10):e0259156. doi: 10.1371/journal.pone.0259156 (PMC8555849; doi:10.1371/journal.pone.0259156)
Supplement: Attachment — Submitted filename: substrate_paper_ReviewerResponse_r2.docx [file pone.0259156.s004.docx]

Comprehensive marine substrate classification applied to Canada’s Pacific shelf

Manuscript # PONE-D-20-40989

Response to follow-up review (Reviewer #2)

2021/09/15

**Summary**

We thank the reviewer for taking the time to consider our revised manuscript. We have addressed all remaining comments as described below. Our responses are indented and italicized below the reviewer’s comments.

**Follow up comments (Reviewer #2)**

I still find the Introduction to be unnecessarily long, containing some material that I suggest could readily be condensed by referencing existing studies. The length of the Introduction is probably something for the editor to decide at this point but there are also a few points I find rather condescending, as currently worded. For instance, in the 'Model performance' section, I find dismissive generalisations such as "... metrics have evolved little ... with most studies continuing to report Cohen's Kappa ...", "adoption of improved metrics has been glacial ...", and "There is a persistent misconception about how to interpret model performance..." to be overly generalised (I am not a geologist but Kappa is very rarely used in relation to predictive model performance in the literature I am familiar with), didactic, and unnecessary.

*We reduced the introduction by about 10% (from 2,199 to 1,902 words) and softened some of the language regarding the shortcomings of model testing. However, we have not removed it entirely because this is a critical challenge for the uptake of such models by management, and is reflected in the cited literature. We have also re-phrased numerous points making them more specific to the material at hand and thus less didactic.*

I also still find the presentation of so many performance metrics to be more confusing than useful, for the most part. Indeed, while trying to interpret the results here I reflected that this illustrates one very good reason why a more refined set of metrics is "commonly provided" in most published studies; practicality of interpretation. With the slightly revised focus in the title, however, (i.e., the method taking priority over the application) there is an argument for inclusion of more metrics.

*We understand that the use of a diverse collection of metrics complicates interpretation. However, multiple metrics are desirable to fully understand model performance (see references in our* Measuring performance *section). Further, error assessment has the potential to guide model improvements, something that accuracy tests cannot do. We have slightly revised the opening sentences of the* Measuring performance *section of the* Discussion *to re-enforce this idea.*

A few minor comments:

116-117: As worded here, I don't see why it would follow that "higher resolution models would perform better in shallow waters" (which I interpreted as meaning that a high resolution model would work better in shallow water than it would in deeper water). I would guess the intended meaning might be worded as "higher resolution models would perform better than coarser resolution models in shallower waters."?

*We have added the suggested edit.*

Line 226: "class weights" is unexplained, as yet, and therefore uninterpretable here.

*We introduce class weighting as part of our first question in the introduction (line 91). We have changed this one occurrence of “class weights” to “class weighting” for consistency.*

Table 2: first column rows are not aligned with others? And "DEMs" is not defined.

*The table is structured to show the difference data sources used for the different types of predictor used, and their native resolutions. We have restructured the table and removed unnecessary information to make the connection between predictors and source data clearer. We have also revised the caption to more clearly describe our intent.*

*We have replaced DEM with ‘elevation model’.*

Line 263: "cross-walked" is a term I've not seen before and only makes sense once you go to Table S1.

*We have replace ‘cross-walked’ with ‘re-classified’.*

Table 3: Caption does not include "imbalance".

*We have added imbalance to the caption.*

Line 474: Why "not unexpectedly"? If we think our models perform well, why would we not 'expect' them to perform equally well against independent data. Suggest there is no need for this in the sentence and, if retained, the expectation should be supported by references (there are a few recent papers on this subject).

*Not unexpectedly because, as described in the introductory section on model performance, model predictive power can be expected to be poorer than model fit. We have re-written the sentence for clarity as: “Not unexpectedly, model forecast skill was generally lower and more variable than model fit.”*
